# Implications of artificial intelligence in periodontal treatment maintenance: a scoping review

**DOI:** 10.3389/froh.2025.1561128

**Published:** 2025-05-14

**Authors:** Raafat Musief Sarakbi, Sudhir Rama Varma, Lovely Muthiah Annamma, Vinay Sivaswamy

**Affiliations:** ^1^ Department of Clinical Sciences, Ajman University, Ajman, United Arab Emirates; ^2^ Center for Medical and Bio-allied Health Sciences Research, Ajman University, Ajman, United Arab Emirates

**Keywords:** artificial intelligence, AI, periodontal maintenance, periodontology, periodontal treatment

## Abstract

Gingivitis and periodontitis, are widespread conditions with diverse influence on oral and systemic health. Traditional diagnostic methods in periodontology often rely on subjective clinical assessments, which can lead to variability and inconsistencies in care. Imbibing artificial intelligence (AI) facilitates a significant solution by enhancing precision metrics, treatment planning, and personalized care. Studies published between 2018 and 2024 was conducted to evaluate AI applications in periodontal maintenance. Databases such as PubMed, Cochrane, Web of Science and Scopus were searched using keywords like “artificial intelligence,” “machine learning,” and “periodontitis.” Studies employing AI for diagnosis, prognosis, or periodontal maintenance using clinical or radiographic data were included. Deep learning algorithms such as convolutional neural networks (CNNs) and segmentation techniques were analyzed for their diagnostic accuracy. AI demonstrated superior performance in detecting periodontal conditions, with accuracy rates surpassing 90% in some studies. Advanced models, such as Multi-Label U-Net, exhibited high precision in radiographic analyses, outperforming traditional methods. Additionally, AI facilitated predictive analytics for disease progression and personalized treatment strategies. AI has transformed periodontal care, offering accuracy, personalized care, and efficient workflow integration. Addressing challenges like standardization and ethical concerns is critical for its broader adoption.

## 1 Introduction

Periodontal diseases, encompassing conditions like gingivitis and periodontitis, are among the most prevalent chronic diseases globally, affecting both oral and systemic health. With periodontitis recognized as the sixth-most common disease worldwide, its implications extend beyond oral discomfort, contributing to systemic diseases such as cardiac conditions, endocrine-related, and possibly pregnancy-related outcomes (1). Early diagnosis and personalized treatment have become critical in managing these conditions effectively, but traditional diagnostic approaches, often reliant on manual assessments and subjective interpretation, present notable limitations (2).

While foundational, traditional treatment methods in periodontology present a range of limitations that compromise their effectiveness. These approaches, such as manual probing for pocket depths and radiographic imaging, often pose significant subjectivity and variability. Measurements can vary between clinicians due to differences in technique, such as probing pressure or angulation, leading to inconsistent results (2). Radiographic interpretation, too, is prone to subjective biases, with subtle indicators of disease progression often being overlooked, particularly in the early stages (3). This variability hinders the reliability of traditional diagnostic practices and undermines their ability to provide consistent care. Furthermore, traditional methods frequently struggle to detect periodontal diseases in their nascent stages. Changes in bone density or tissue structure are often too subtle for conventional tools to identify, allowing the disease to progress unchecked and reducing the effectiveness of subsequent interventions (1).

Incorporating AI into periodontology effectively addresses numerous challenges, transforming diagnosis with precision, efficiency, and adaptability. AI-powered tools eliminate operator variability by standardizing measurements and analyses, enhancing diagnostic consistency across clinicians. This consistency improves diagnostic reliability and ensures that patients receive uniform care and maintenance regardless of the provider. Furthermore, AI excels in early detection by identifying subtle patterns and anomalies that traditional methods might overlook. AI systems can analyze radiographs and intraoral scans to detect minor bone density or tissue integrity changes, facilitating earlier interventions that improve patient-centric outcomes (3).

AI in periodontics holds the potential to drive a paradigm shift by addressing these challenges through enhanced precision, predictive analytics, and personalized treatment strategies. By leveraging machine learning (ML) algorithms, computer vision, and data-driven models, AI enables clinicians to identify subtle patterns and predict disease progression, facilitating earlier and more targeted interventions to focus on patient care and alleviating administrative burdens (3, 4).

One of the most transformative applications of AI in periodontology is its capacity to integrate diverse datasets, providing a holistic assessment of a patient's oral health. AI provides tailored risk assessments and personalized treatment plans by analyzing clinical records, imaging data, and demographic information (4). This capability aligns with the goals of precision medicine, emphasizing proactive, individualized care.

Moreover, AI's role extends to personalized treatment, leveraging patient-specific data to develop tailored risk assessments and treatment plans. Machine learning models analyze demographic, lifestyle, and genetic information to predict disease susceptibility and progression, enabling proactive interventions (5). Using AI in predictive analytics also facilitates resource optimization, guiding clinicians in prioritizing high-risk patients and minimizing unnecessary procedures (4).

**Figure 1:**
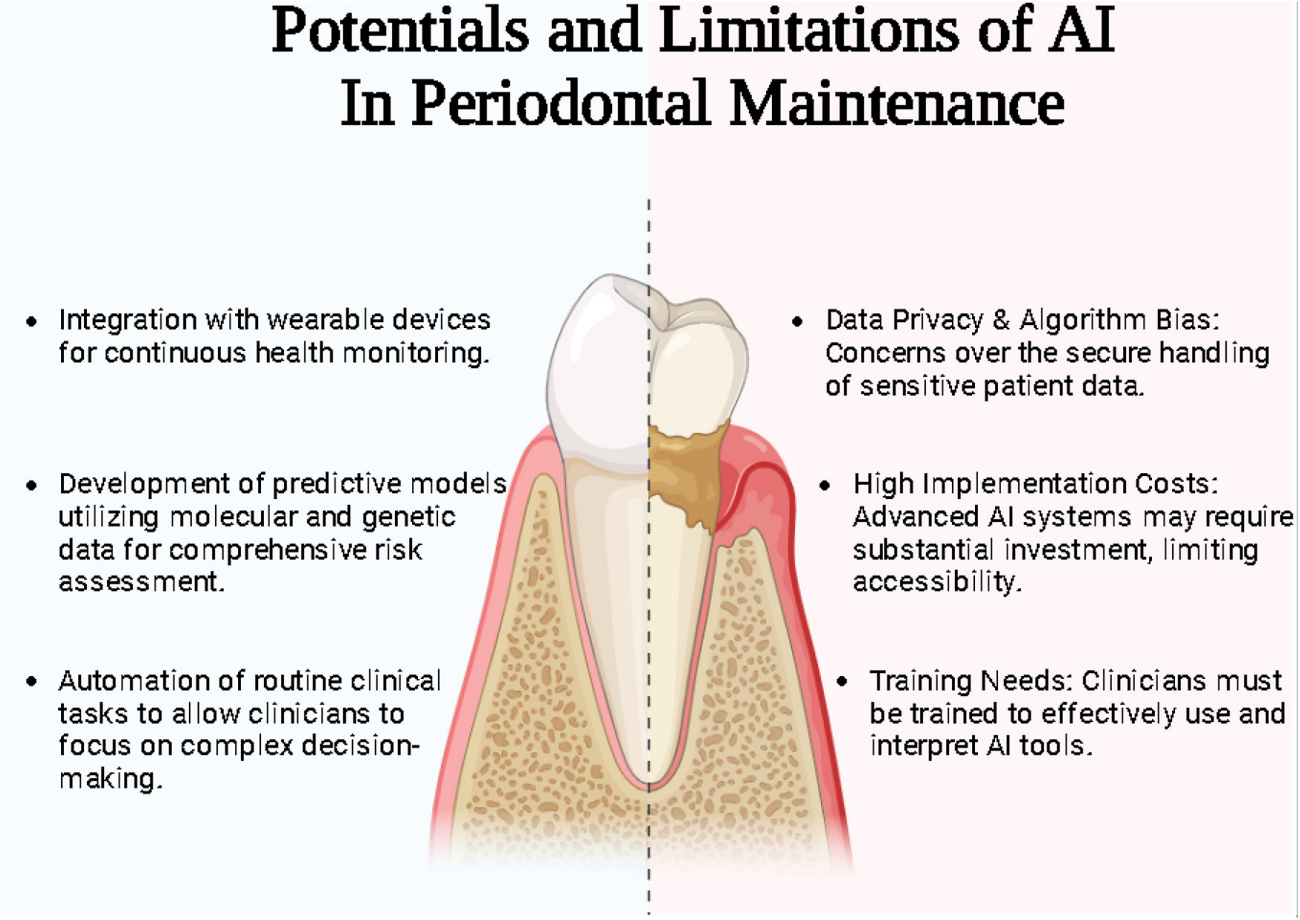
Potentials and limitation of AI in periodontal maintenance.

Despite these advancements, significant challenges persist, particularly in standardizing methodologies and initiating transparent algorithms and data privacy environments (1). The heterogeneity of reporting outcomes in AI research further underscores the need for unified frameworks to ensure comparability and reproducibility across studies (2) (Figure 1).

Beyond diagnostics, AI's integration into periodontal maintenance enables the development of highly personalized care strategies. By analyzing complex patient data—encompassing genetic profiles, clinical records, and lifestyle factors—AI offers tailored risk assessments and treatment plans that align with the principles of precision medicine. This shift towards individualized care is more effective and fosters proactive management, reducing the incidence and severity of periodontal diseases over time. Additionally, AI's efficiency in automating data analysis and streamlining clinical workflows ensures that periodontal maintenance is both cost-effective and accessible (6).

### 1.1 Current challenges in periodontal diagnostics

Despite advancements in dental technology, diagnosing periodontal disease accurately and efficiently remains a challenge. Some of the key issues faced in periodontal diagnostics include.

#### 1.1.1 Subjectivity in periodontal disease assessment

Routine periodontal diagnosis involves clinical examinations, probing pocket depths, bleeding on probing (BOP), and radiographic analysis. However, these methods are often subjective, and results can vary depending on the clinician's experience and technique (7).

#### 1.1.2 Limitations of probing depths

Periodontal probing, the gold standard for assessing pocket depth, is prone to errors due to variations in probing force, angulation, and patient discomfort. Additionally, it does not provide real-time insights into disease activity or predict future progression (8).

#### 1.1.3 Periodontal disease early detection challenges

Most periodontal diagnostic tools detect the disease only after significant tissue destruction. There is a lack of reliable biomarkers or imaging techniques to identify periodontal disease in its earliest stages before irreversible damage occurs (9).

#### 1.1.4 Radiographic limitations

Conventional dental x-rays help detect bone loss but do not provide information on soft tissue health or ongoing inflammation. Advanced imaging techniques like CBCT (Cone Beam Computed Tomography) offer better visualization but are costly and not always accessible (10).

#### 1.1.5 Lack of molecular diagnostics

The role of biomarkers in periodontal diagnosis has been studied extensively, and its importance in diagnosis, prognosis, and providing positive treatment outcomes has been highlighted in the literature. Nevertheless, incorporating it into routine clinical use is a challenge (11).

#### 1.1.6 Patient compliance

Patient compliance is the most important factor detrimental to the overall outcome. Periodontal treatment can have positive outcomes only if patients are educated and motivated. Lack of awareness leads to poor compliance, making early detection and management difficult (12).

By addressing existing challenges and identifying pathways for integration, the discussion seeks to demonstrate how AI can elevate periodontal care in terms of precision, efficiency, and patient-centered outcomes.

### 1.2 Applications of artificial intelligence in periodontal maintenance

Despite advancements such as the updated *2017 AAP and EFP Classification of Periodontal Diseases*, which enables more precise evaluation of periodontal conditions through alveolar bone loss metrics (13). Traditional reliance on subjective clinical assessments and time-consuming radiographic analyses creates diagnostic accuracy and consistency gaps (14). Effective periodontal maintenance is vital for halting the progression of periodontal diseases and preserving both oral and systemic health. Regular maintenance involves professional interventions such as scaling, root planning, and continuous monitoring of oral hygiene. These practices aim to control plaque accumulation and inflammation, primarily contributing to progression from gingivitis, a reversible condition, to periodontitis, a chronic and potentially debilitating disease (4, 15).

### 1.3 Concomitant applications of AI

Furthermore, advanced AI diagnostic tools are revolutionizing the field, offering improved accuracy and early detection of periodontal changes. For instance, a recent retrospective study by Kim et al. using a deep learning-based method called DeNTNet reported 73.4% to 99% accuracy rates for evaluating bone loss from images using AI. Additionally, automated models based on periapical radiographs demonstrated accuracy rates as high as 99% in staging severe radiographic bone levels, highlighting AI's proficiency in identifying advanced periodontal conditions (16). These advancements allow clinicians to offer proactive care, reducing the reliance on invasive treatments (11). Similarly, Chang et al. reported an accuracy of 0.93 for bone detection and 0.91 for identifying the cementoenamel junction (CEJ) and teeth, demonstrating AI's capability to perform complex diagnostic tasks with high reliability (17).

Routine periodontal maintenance is associated with broader health benefits. It helps mitigate systemic inflammation linked to cardiovascular diseases and diabetes (1). This underscores the significance of integrating periodontal care as a component of overall health management, emphasizing the value of consistent, personalized, and technology-supported maintenance strategies. AI's contribution extends beyond diagnosis by supporting personalized treatment plans. AI facilitates this by integrating genetic, clinical, and lifestyle data to tailor interventions to individual patient needs. Artificial intelligence in dentistry primarily leverages machine learning (ML), deep learning (DL), and computer vision. These technologies are employed for predictive analytics, diagnostic assistance, and treatment planning. CNNs, a subset of DL, are particularly effective in analyzing radiographic images and detecting patterns indicative of periodontal disease (18). For example, Krois et al. evaluated periodontal bone loss with an accuracy of 0.81 using CNNs, while (Rini Widyaningrum et al. achieved even greater accuracy at 95% using advanced segmentation techniques. These tools reduce variability among clinicians, ensuring consistent and reliable outcomes (18, 19).

AI applications span several domains in dentistry, including radiographic analysis, where algorithms detect early signs of disease, such as bone loss, with high precision, thereby reducing diagnostic variability among clinicians (20). Additionally, AI facilitates the preparation of personalized treatment plans by evaluating patient data to customize interventions according to risk profiles and disease-related characteristics (21, 22).

AI could become a significant game changer in periodontal maintenance, providing innovative solutions across diagnostic assistance, disease progression prediction, personalized treatment planning, and patient monitoring. AI-powered tools, such as convolutional neural networks (CNNs), enhance diagnostic accuracy by analyzing imaging data and clinical parameters to detect early signs of periodontal diseases like alveolar bone loss and periodontal pockets with greater consistency and reliability than manual methods (19). Predictive modeling using advanced machine learning techniques, such as Random Forest and Support Vector Machines, has demonstrated exceptional accuracy, often exceeding 95%, in forecasting disease progression based on patient histories and risk factors, thus enabling timely interventions (5). Furthermore, AI supports the creation of personalized treatment plans by integrating genetic, clinical, and lifestyle data, aligning with precision medicine principles to optimize care tailored to individual patient needs (1, 5). Real-time AI-monitoring tools further revolutionize periodontal care by tracking patient adherence to treatment regimens and measuring therapeutic outcomes, allowing clinicians to make timely adjustments to ensure effective and efficient care delivery (6). Collectively, these advancements underline AI's potential to redefine periodontal maintenance by delivering more accurate, personalized, and proactive patient care.

### 1.4 Integration and facilitating data analysis

**Figure 2:**
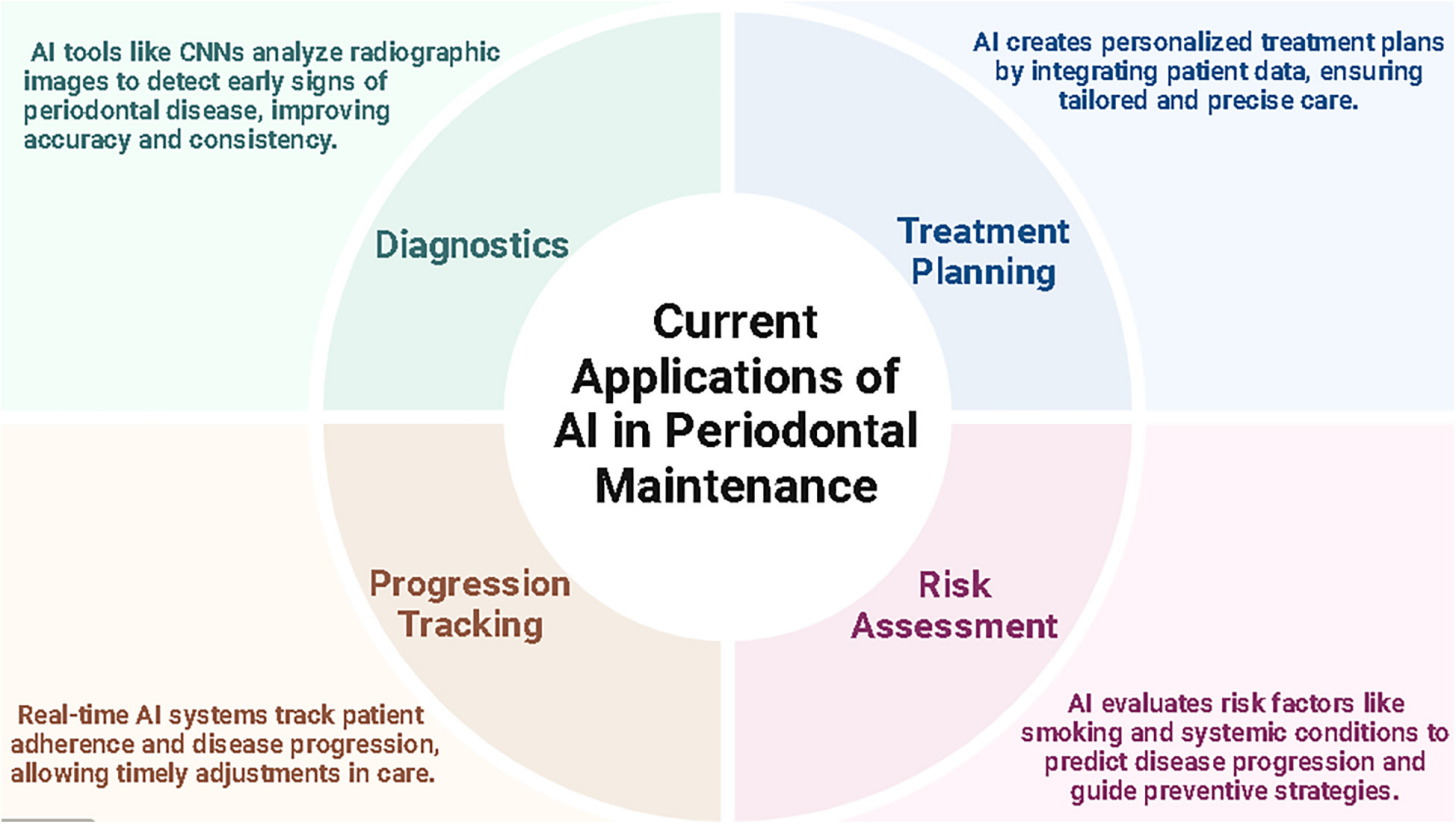
Current applications of AI in periodontal maintenance.

Incorporating AI in periodontal maintenance offers many advantages, significantly improving diagnostic accuracy, treatment efficiency, and scalability in delivering care. Machine learning algorithms and convolutional neural networks (CNNs) enable the rapid processing and analysis of vast datasets, facilitating consistent, evidence-based decisions that enhance patient outcomes. For example, AI-powered diagnostic tools can identify early signs of periodontal disease more precisely than manual methods, reducing variability and error (2). Predictive models also allow clinicians to forecast disease progression and customize treatment plans, leveraging patient-specific data to tailor interventions effectively. According to Amasya et al., an AI-powered diagnostic tool achieved an accuracy of 0.977 and an F-score of 0.948 in analyzing panoramic radiographs, illustrating the potential for precise and automated diagnosis (22) (Figure 2).

AI in periodontal care introduces significant opportunities and ethical challenges, necessitating a careful and balanced approach to its implementation. On the one hand, AI has the potential to transform periodontal treatment planning by improving precision, reducing human error, and personalizing care (23). Additionally, successful integration into clinical practice depends on adequately training dental professionals to understand AI systems, interpret outputs accurately, and incorporate these insights into existing workflows. This requires structured educational initiatives and ongoing support to help practitioners adapt to technological advancements while maintaining high standards of patient care (3). By addressing these ethical and practical considerations, AI can be effectively leveraged to enhance periodontal care while ensuring equity, safety, and transparency.

### 1.5 AI-enabled diagnostic capabilities

A deep learning model developed by Krois et al. enabled the segregation of periodontal bone loss from routine panoramic radiographs with a high success rate. Another study by Balaei et al., based on the convolutional neural network (CNN) model, was used to detect potential gingival bleeding from intra-oral radiographic images (19, 24).

### 1.6 Predictive analysis for disease progression

Though ML and AI models have provided a potential help in automated imaging interpretation, data extraction, clinical risk prediction, and quality enhancement as reported in clinical simulation models based on cardiology, more specifically related to aortic aneurysms as reported in a study by Zargarzadeh et al. (25) In terms of periodontal disease progression, the pathway of inflammation depends on the severity of the inflammation and also to a major extent to patient compliance. Considering these factors, AI and machine learning-enabled models require further evaluation to predict disease progression mechanisms (26).

### 1.7 Personalized periodontal treatment planning

AI-specific algorithms can provide a “Bespoke” generated treatment plan for specific needs, further detailing data specific to individual needs based on periodontal oral parameters, genetic-related markers, and serum, saliva, and GCF-related biomarkers. A study highlighting the possibility of incorporating genetic-related algorithms and fuzzy logic details potential treatment planning related to periodontal therapy (6, 27). Personalized treatment planning incorporating, most importantly, clinical treatment outcomes and adherence to clinical treatment guidelines provides a longitudinal positive outcome and patient satisfaction.

The current review aims to delve into AI's specific applications in periodontal maintenance, examining its current achievements and potential to revolutionize periodontal care practices.

## 2 Materials and methods

### 2.1 Research questions

1. What is the updated role of Artificial Intelligence related to periodontal disease?
2. Does Artificial Intelligence play an important role in periodontal maintenance?
3. What potential outcomes exists by incorporating Artificial Intelligence in periodontal maintenance?

### 2.2 Eligibility criteria and study selection

Inclusion Criteria

• Studies focused on the role of Artificial Intelligence in periodontal disease.
• Studies providing data on synthesis and modulation of AI to periodontal health.
• Peer-reviewed articles, observational studies-retrospective and prospective, & clinical trials.
• Articles in English to ensure consistent analysis and accessibility.

Exclusion Criteria

• Studies not directly addressing periodontal disease or irrelevant to AI.
• Publications focusing on non-periodontal diseases and AI.
• Studies in languages other than English or without full-text availability.

**Figure 3:**
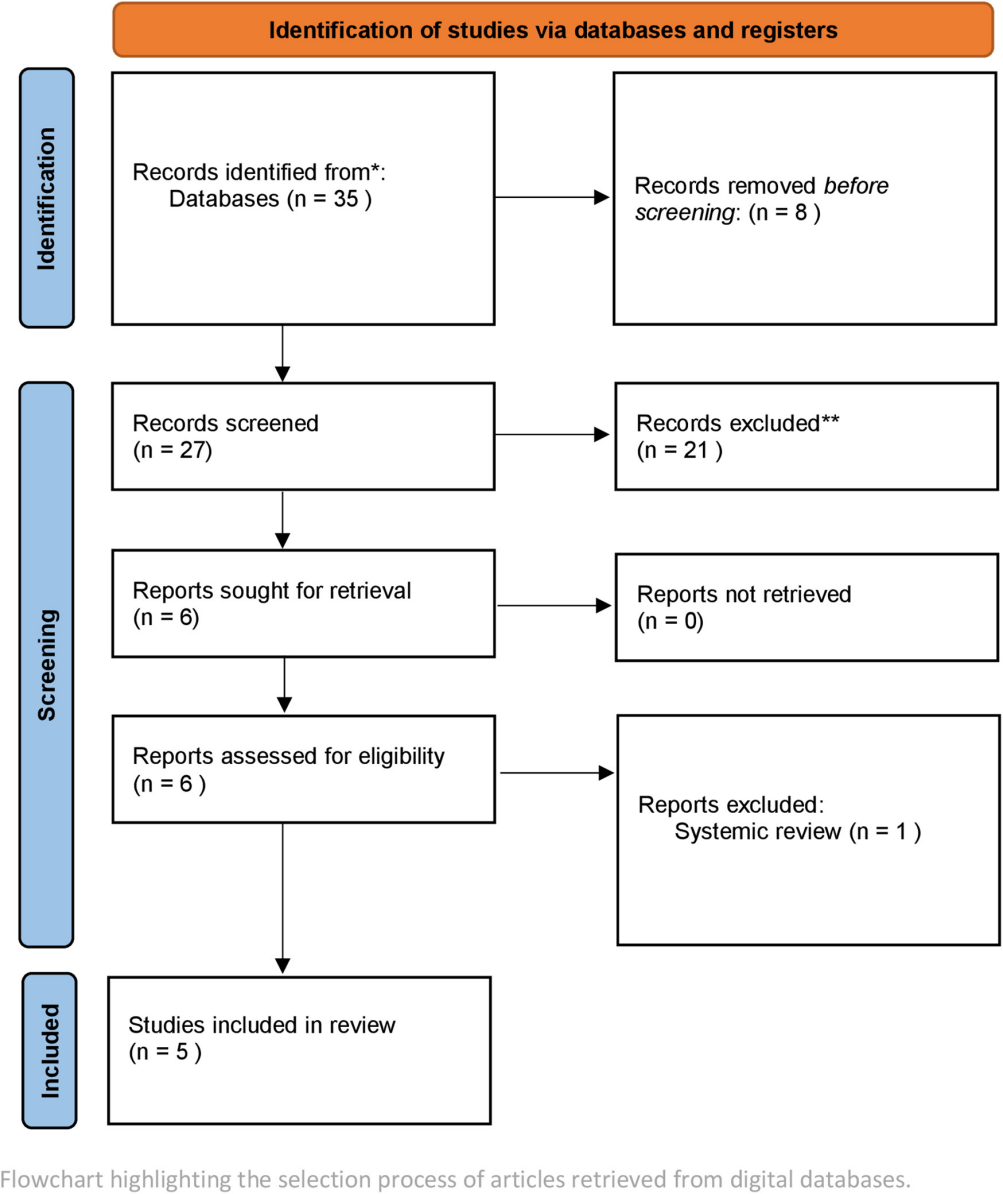
Flowchart highlighting the selection process.

Studies published between 2018 and August 2024 were selected. Four reviewers were involved in the selection of studies. To achieve standardization and to facilitate consensus a double screening method was employed.The search encompassed databases including PubMed, Scopus, Cochrane, and Web of Science. The keywords employed included combinations such as [(artificial intelligence) OR (machine learning)] AND (periodontitis OR periodontal maintenance). Only peer-reviewed articles published in English were included. Inclusion criteria comprised original studies, Experimental studies, that utilized AI for diagnosis, prognosis, or maintenance of periodontitis. Excluded were studies outside English, narrative reviews, and studies solely discussing theoretical AI frameworks without clinical or radiographic data (Figure 3).

The selected primarily evaluated AI applications in diagnosing periodontal diseases, staging and grading periodontitis, and monitoring treatment outcomes. These studies predominantly employed deep learning algorithms, including convolutional neural networks (CNN), Mask R-CNN, and U-Net, which are widely used for radiographic image segmentation and diagnosis of periodontal bone loss. Both panoramic radiographs and periapical radiographs were utilized for AI-based assessments of periodontal conditions. Studies were further analyzed to evaluate the accuracy of AI-based systems in comparison with conventional clinical diagnosis.

## 3 Results

### 3.1 Study selection and data compilation

The study selection is outlined in Figure 3. A total of 35 articles were identified after screening and removal of duplicates. A total of 30 articles were excluded after not meeting the inclusion criteria. Most of these excluded studies tested software or used algorithms for broader diagnosis of oral diseases rather than specifically identify periodontal architecture and its changes.

### 3.2 Data extraction for quality & bias

Quality and possible bias for diagnostic accuracy studies was looked for using APPRAISE-AI was employed to assess study quality (28). Furthermore, From the chosen papers, the two reviewers separately gathered pertinent data, which was subsequently evaluated for quality. The study design, sample size, and methodological rigor were all evaluated as part of the quality assessment. Cohen's Kappa coefficient (K = 0.80), which measures inter-reviewer agreement, showed a high degree of agreement. A senior reviewer was consulted in order to settle disagreements during the study selection process. Citations were arranged and maintained using reference management software (EndNote version X9).

### 3.3 Datasets

The datasets ranged from 100–12,179 with a mean of 4,126 for solely panoramic radiographs and 474 for periapical. Only one study used teeth to evaluate prognosis based on AI. It is worth noting that in most of the studies using retrospective study design, the inference related to AI was significantly higher than manual observation done by clinicians for identifying periodontal bone loss.

### 3.4 AI employed architectures

The results from the selected studies demonstrated AI significantly enhances the accuracy of detecting periodontal bone loss (PBL) on radiographic images. For instance, an automated deep learning method achieved an accuracy of 85%–91% in diagnosing periodontal bone loss and staging periodontitis compared to clinicians' assessments (17). The study reported that the AI based tool could help dentists monitor and evaluate periodontitis on panoramic radiographs in a more systematic and efficient way (17). A CNN-based system demonstrated a 10.69% margin of error for measuring PBL, simulating current inter-observer variations and highlighting the precision and reliability of AI-based methods in periodontal evaluations (20).

AI-based segmentation techniques, such as Multi-Label U-Net and Mask R-CNN, were evaluated for panoramic radiograph analysis (18). These findings emphasize the ability of AI models to accurately analyze radiographic images and delineate periodontal structures, which is critical for early disease detection and intervention. The scores were higher and further demonstrated Multi-Label U-Net was superior in image segmentation compared to Mask R-CNN (18). The study by Chang et al. involved a deep learning hybrid method. The method was made to individually assess bone loss in each individual tooth. The deep learning method was used to detect radiographic bone level-RBL, for the whole complement of teeth from orthopantomography-OPG. The percentage bone loss was further calculated using the tooth length which complemented the staging criteria used for the 2017 Classification of periodontal and Peri-implant Diseases and conditions (13). This hybrid model demonstrated superior diagnosis of periodontal bone loss and further helped in staging the condition with better reliability and accuracy (17).

A deep learning-based method developed by Kim et al, detected periodontal bone loss using automated diagnostic support system employing panoramic radiographs. A method called DeNTNet (Deep Neural transfer network) was used for this study. The tool was initially validated using 12,179 panoramic radiographs. The study concluded a higher score of 0.75 when compared to clinician score of 0.69 (16).

Prognosis of tooth and associated structures were evaluated in a study by Lee et al. The study used three AI based machine learning methods-gradient boosting classifier, decision tree classifier and random forest classifier to develop an algorithm. The study inferred that the decision tree classifier scored the highest (0.8413) compared to the other methods in the decision-making process further highlighting the implementation of AI in decision making related to tooth prognosis.

The studies selected have used different learning methods incorporating both spatial and descriptive analysis. The tools have employed algorithms to develop a significant robust model that can possibly help reason, comprehend and navigate through confounding variables. The studies though used mostly in retrospective data analysis have incorporated deep learning and semantic segmentation to evaluate periodontal bone loss. It offers better reliability and accuracy when compared to conventional tools such as CAD and other radiographic tools. The inferences from most of the studies highlighted the possible use of a web-based AI software in detecting periodontal bone loss on radiographic images (29).

## 4 Discussion

AI has also demonstrated the ability to predict outcomes for compromised teeth, providing valuable insights for treatment planning. A deep learning-based CNN model achieved a predictive accuracy of 82.8% for premolars and 73.4% for molars, offering a robust method for prognosis and extraction decisions in clinical practice (30). These results indicate that AI can play a significant role in determining tooth prognosis, particularly in cases where human assessment may be less consistent or subjective. A systematic review further found that AI models, including deep CNNs, provide comparable accuracy to experienced clinicians in classifying periodontal diseases. However, performance varied due to differences in datasets and algorithm optimization, indicating the need for standardized approaches to improve reliability and reproducibility across studies (31) (Table 1).

**Table 1:** List of studies using AI for diagnosing periodontal conditions.

Author	Year	Study Type	Results
Chang et al.	(17)	Retrospective study	Accuracy of 85%–91% in diagnosing periodontal bone loss and staging periodontitis.
Kim et al.	(16)	Retrospective study	Deep neural transfer network DeNTNet was superior with a F1 score of 0.75 compared to clinician score of 0.69
Rini Widyaningrum et al.	(18)	Retrospective study	Multi-Label U-Net achieved Dice coefficient of 0.96 and IoU score of 0.97, outperforming Mask R-CNN.
Amasya et al.	(20)	Retrospective study	Cohen's kappa coefficient of 0.96 for PBL detection; AI tools improved diagnostic consistency.
Lee et al.	(35)	Retrospective study	Predictive accuracy of 82.8% for premolars and 73.4% for molars for tooth prognosis.

Automated tools utilizing AI have also shown promise in reducing inter-observer variability in periodontal assessments. One study reported a Cohen's kappa coefficient of 0.96 for periodontal bone loss detection, reflecting near-perfect agreement between AI predictions and expert evaluations (22, 32). Such tools not only improve diagnostic consistency but also offer clinicians a reliable second opinion for more accurate and objective evaluations. Additionally, AI systems can facilitate personalized treatment strategies by integrating clinical and molecular data, promoting precision periodontal care (1). On the other hand, the possibility of heterogeneity and limited quality of reporting in the studies also underpins the limitations in generalizing AI based evaluations when looking at diverse populations and different datasets. The possibility of false positives and negatives depending on the volume of the sample size relates to this variability. Larger data or larger sample size provides more precise algorithms (33).

The implementation of AI-assisted tools, such as web-based software for continuous monitoring of radiographic bone loss, has further shown promise in maintaining periodontal health. These systems provide efficient and cost-effective periodic assessments, enabling early detection of bone loss and disease recurrence. By integrating AI into regular maintenance strategies, clinicians can optimize treatment outcomes and prevent the progression of periodontal disease (1, 5, 22, 33).

A combination of AI-based detection and conventional CAD approaches has demonstrated high reliability for diagnosing periodontal bone loss and classifying disease severity. One study reported a Pearson correlation coefficient of 0.73 (*p* < 0.01), underscoring the potential of hybrid methods to enhance diagnostic accuracy and improve clinical workflows (18). These approaches integrate the strengths of both AI and traditional methods, offering a comprehensive solution for periodontal disease management (34).

Several studies highlighted AI's potential in treatment monitoring and disease recurrence prediction. AI-powered platforms provided real-time feedback on disease status, enabling clinicians to track patient progress and adjust treatment plans accordingly (32, 33, 35).

These findings collectively emphasize the possible efficacy of AI in periodontal maintenance, particularly through automated bone loss detection, precise image segmentation, and enhanced diagnostic accuracy (Table 1). AI tools demonstrate significant potential to support clinicians in maintaining periodontal health through early intervention and consistent monitoring.

### 4.1 Current diagnostic gaps and limitations

ML and DL, offers the capability to enhance diagnosis, treatment planning and provide personalized periodontal treatment care and maintenance. AI-powered radiographic analysis has demonstrated significant improvements in detecting periodontal conditions such as bone loss and periodontal pocket formation, surpassing the limitations of traditional subjective diagnostic methods. These systems provide consistent, objective evaluations, enabling earlier and more accurate diagnoses. Similarly, predictive AI models can forecast disease progression and response to treatment, empowering clinicians to adopt proactive and targeted therapeutic strategies that optimize patient outcomes (34).

Despite these related advancements, challenges persist in the implementation of AI in periodontics. A critical issue is the lack of standardized methodologies for data collection, model training, data heterogeneity, lack of standardization, and ethical concerns regarding patient data usage and performance validation. This variability limits the generalizability of AI applications across diverse clinical settings. Furthermore, biases in training datasets, stemming from underrepresentation of specific populations, risk perpetuating healthcare disparities. Ethical and legal concerns, such as ensuring data privacy, patient consent, and algorithm transparency, require robust frameworks to establish trust and accountability in AI systems (35). Addressing these diverse challenges will necessitate collaboration between various stakeholders such as researchers, clinicians, and policymakers to establish comprehensive standards, safeguard patient interests and furthermore require nuanced socio-political comprehension.

### 4.2 Ethical, technical and regulatory challenges

Ethical concerns and data privacy challenges are significant barriers to AI adoption in healthcare, particularly in predictive analytics. As AI usage in healthcare expands, key issues include protecting personal data, preventing patient injustice, and ensuring accountability. Since AI processes patient information, maintaining data privacy is crucial for trust and regulatory compliance. Safeguarding patient data involves incorporating robust security measures to mitigate unauthorized access, ensuring secure storage, and establishing clear data management protocols. Regulations like HIPAA and GDPR are essential for maintaining compliance and fostering patient trust (36). Given AI's inherent ability to learn and adapt, substantial regulatory challenges must be confronted. Innovative solutions are necessary to guarantee both efficacy and safety.

The FDA is now evaluating regulatory frameworks that allow for the gradual improvement of AI algorithms while upholding stringent safety and efficacy standards. The regulation of ever-growing AI systems is a considerable problem in this regard. Conventional regulatory frameworks are inadequate for overseeing the swift advancement of artificial intelligence technology. The FDA has included a “predetermined change control plan” in its regulation suggestions, permitting manufacturers to alter AI algorithms after approval. Artificial Intelligence in healthcare includes many technological advancements that empower Large Language Models (LLMs) to do tasks such as learning, problem-solving, and decision-making, which traditionally need human intelligence. Mitigating AI risks in healthcare requires the development of comprehensive frameworks. These frameworks must integrate sociological, legal, and ethical considerations alongside technological ones (37).

The FDA plans to support initiatives that tackle health disparities associated with AI use in medical product development such as radiological equipment, leveraging existing diversity, and equity. This method reduces biases in AI applications by utilizing representative and diverse datasets (36, 37).

Furthermore, the FDA emphasizes the imperative of ongoing supervision of AI tools in healthcare. This focus on maintaining adherence to standards and assuring performance and reliability across the AI lifecycle underscores the imperative of continuous monitoring in reducing AI risks in healthcare.

Another ethical challenge is implicit bias, which arises when AI is expected to replace human doctors in making medical and ethical decisions. AI systems can unintentionally introduce biases, leading to disparities in healthcare outcomes for certain patient groups. To prevent this, AI development must prioritize fairness and equity in algorithm design to avoid exacerbating existing healthcare inequalities. Accountability is another critical ethical concern in AI implementation. Determining responsibility for AI-related errors is challenging, as AI systems often integrate human expertise and decision-making. For AI to function safely in healthcare, clear accountability structures and effective error detection and correction mechanisms must be established. Addressing these ethical issues allows healthcare providers to enhance care quality and efficiency through AI-driven predictive analytics while maintaining patient autonomy and privacy (38, 39).

Ethical concerns, particularly around data privacy, algorithm transparency, and biases in training datasets, pose significant hurdles. Additionally, the high cost of implementation, resistance to technological change among practitioners, and the lack of standardization in AI methodologies hinder seamless integration into clinical workflows (4). Addressing these challenges requires robust regulatory frameworks, interdisciplinary collaboration, and comprehensive training programs to ensure that AI is utilized ethically and effectively.

Integrating AI further into routine dental practices involves significant costs, which may take considerable time to break even. Additionally, dentists may encounter challenges such as staff education, patient awareness, and acceptance when marketing this tool in daily practice. Another crucial factor to consider is the need for periodic training to upgrade existing tools, which will require additional time and investment.

## 5 Potential future roadmap

Future research directions must prioritize the validation and refinement of AI models across geographically and demographically diverse populations. This includes developing algorithms capable of processing multimodal data, such as clinical records, imaging, and genetic information, to deliver holistic and individualized periodontal care. Additionally, integrating AI into clinical workflows demands the creation of user-friendly platforms that provide real-time decision support without disrupting routine practice. Such innovations will not only improve clinicians' efficiency but also enhance the scalability and adaptability of AI-driven solutions in periodontal management and furthermore mitigate the limitations that currently using AI in periodontal treatment are seen.

Addressing data heterogeneity and bias can be addressed by developing participant targeted AI algorithms, responsible data sharing, this to an extent facilitates interoperability and code sharing to allow AI algorithms in synthesizing underrepresented data. These steps can provide improved periodontal treatment maintenance regimes and facilitate positive periodontal treatment outcomes (40).

The broader implications of AI in periodontics extend beyond individual care to the systemic enhancement of dental practice. By facilitating clinical diagnosis and assisting in routine procedures, AI reduces the clinical burden on practitioners. Furthermore, AI has the potential to bridge disparities in periodontal care delivery by providing remote diagnostic and treatment planning capabilities, particularly in underserved or resource-limited regions. It has the potential to uncover surprising correlations and relationships that could be remain unidentified in human driven analysis, it can furthermore provide new insights in periodontal maintenance by analyzing data from variables such as biomarkers, social determinants, and environmental exposure. It has the potential to uncover novel correlations and better periodontal treatment delivery and maintenance by challenging current paradigms and highlighting new areas for intervention (41).

## 6 Conclusion

By enhancing diagnostic accuracy, facilitating personalized care, and addressing systemic healthcare challenges, AI promises to reshape periodontal practice. However, realizing this potential requires addressing key challenges, fostering innovation, and cultivating a culture of collaboration. The integration of AI into periodontology not only offers a pathway to precision medicine but also contributes to improved systemic health, quality of life, and equitable care delivery, marking a pivotal step forward for modern dental healthcare.
