# Supplementary material for: Implications of artificial intelligence in periodontal treatment maintenance: a scoping review
Source: Front Oral Health. 2025 May 14;6:1561128. doi: 10.3389/froh.2025.1561128 (PMC12116603; doi:10.3389/froh.2025.1561128)
Supplement: Supplementary file 1 [file Table1.docx]

Supplemental Online Content

Kwong JCC, Khondker A, Lajkosz K, et al. APPRAISE-AI tool for quantitative evaluation of AI studies for clinical decision support. *JAMA Netw Open*. 2023;6(9):e2335377. doi:10.1001/jamanetworkopen.2023.35377

**eAppendix.** Supplemental Methods

**eTable 1.** The APPRAISE-AI Tool to Assess Quality of AI Studies in Medicine

**eTable 2.** Scoring Rubric for Expert Ratings for Each Included Article **eTable 3.** APPRAISE-AI Tool on a High-Quality Image Analysis Study **eTable 4.** APPRAISE-AI Tool on a High-Quality Classification Study **eTable 5.** APPRAISE-AI Tool on a High-Quality Survival Analysis Study

This supplemental material has been provided by the authors to give readers additional information about their work.

**eAppendix.** Supplemental Methods Sample size calculations

All sample size calculations were determined using PASS version 15.0.5 (NCSS, LLC. Kaysville, Utah, USA).

*Spearman’s rho*

A sample size of 28 achieves 82% power (95% CI 81-83%) to detect a Pearson correlation of 0·53 using a two-sided hypothesis test with a significance level of 0·05. These results are based on 10000 Monte Carlo samples from the bivariate normal distribution under the alternative hypothesis.

*Intraclass correlation coefficient*

A sample size of 28 studies with 2 observations per study achieves 81% power to detect an intraclass correlation coefficient of 0·45 under the alternative hypothesis when the intraclass correlation coefficient under the null hypothesis is 0 using an F-test with a significance level of 0·05.

**eTable 1.** The APPRAISE-AI Tool to Assess Quality of AI Studies in Medicine

For items that indicate “select one of the following”, select the value that yields the higher possible score where applicable. For example, if a study provided both internal and external validation, a score of 3 should be recorded for Item 8: Method of evaluating model generalizability. All other items are considered “select all that apply”. For example, if a study included data from multiple countries (+1) and community hospitals (+2), a score of 3 would be assigned for Item 4: Setting of institutions. The overall APPRAISE-AI score was graded as follows: very low quality, 0 – 19; low quality, 20 – 39; moderate quality, 40 – 59; high quality, 60 – 79; very high quality, 80 – 100.

| **Item** | **Description** | **Score** | **Total** |
| --- | --- | --- | --- |
| **Title** | | | |
| 1 | **Title**  Identify the report as an AI application to a specific clinical question. | + 1 | /1 |
| **Introduction** | | | |
| 2 | **Background**  Describe the clinical problem and rationale for developing AI models.  Review existing relevant literature exploring AI models for the problem being addressed. | + 1 | /1 |
| 3 | **Objective and problem**  Clearly state what the proposed AI model(s) aims to address with respect to study population and outcome. | + 1 | /1 |
| **Methods** | | | |
| 4 | **Source of data**  Describe how the dataset was obtained (e.g., single/multi-center, local/national database, etc.), and study period. If relevant, the diversity of the dataset is also described (e.g., inclusion of community hospitals, low/middle income populations, and institutions from other countries) | Number of institutions, select one of following   - Single institution: + 0 - Multiple institutions (>1): + 2   Study period and length of follow-up (if applicable), select one of following   - Not reported: 0 - Reported: + 1   Setting of institutions, select all that apply   - Academic institutions: + 0 - Institutions from multiple (>1) countries: + 1 - Community-based or rural hospital(s): + 2 - Low/middle income patient populations: + 2 | /8 |
| 5 | **Eligibility criteria**  Specify all criteria for inclusion/exclusion of patients and features. Provide appropriate details (e.g., adults, age > 18) and rationale | - Inclusion criteria: + 1 - Exclusion criteria: + 1 - Details and rationale for criteria provided: + 1 | /3 |
| 6 | **Ground truth**  Define the ground truth of interest. Describe how it was collected (e.g., manual annotation by experts) and encoded (e.g., binary, categorical, dichotomized continuous, continuous variable, etc.). For unsupervised learning, describe what measure(s) and associated data will be used to assess cluster validity (e.g., correlating disease-specific features with overall survival). | Ground truth is clearly defined, select one of the following   - Yes: + 2   - No: + 0  Quality of ground truth, select one of the following   - Single, non-expert: + 1 - Single, expert: + 2 - Multiple (>1), non-experts (e.g., crowd-sourced): +2 - Multiple (>1), experts: + 4 - Objective, well-captured ground truth (e.g., in-hospital mortality): + 4 | /6 |

| **Item** | **Description** | **Score** | **Total** |
| --- | --- | --- | --- |
| 7 | **Data abstraction, cleaning, preparation**  Describe the methods used to develop the final dataset, with consideration of the following:   - Feature abstraction - Handling of missing data (e.g., removal, imputation) - Feature engineering - Removal of features | Feature abstraction   - Rationale provided for choice of candidate features (e.g., based on prior research, clinical relevance, available data, etc.): + 1 - Time-windows for abstracted features are specified (e.g., vital signs recorded within the past 12 hours will be used to predict sepsis): + 1   Handling of missing data, select one of the following   - Unclear if there is missing data or how it was handled: + 0 - Removal of samples with missing data (i.e., complete case analysis): + 0 - Explicit modeling of missing data without justification: + 1 - Explicit modeling of missing data with appropriate justification (e.g., directly through AI model, multiple imputation, other statistical approaches): + 2 - Clear statement that there is no missing data: + 2   Feature engineering   - Transformation/Augmentation: Details provided for how data was altered to change its representation (e.g., normalization, log-transformation, one-hot encoding, image rotation, image translation, adjusting image contrast). If not performed, it should be explicitly stated: + 1 - Modification/Cleaning: Details provided for how data was altered in a non-uniform manner (e.g., outlier removal). If not performed, it should be explicitly stated: + 1   Removal of features   - Method reported (e.g., clinical judgement, principal component analysis, recursive feature elimination, correlation, or ablation analysis). If not performed, it should be   explicitly stated: + 1 | /7 |
| 8 | **Data splitting**  Specify how the data was divided into the training and testing cohorts | Method of data splitting, select one of the following   - Not reported: + 0 - Random split (i.e., random 80:20 train-test split): + 1 - Temporal split (i.e., for a dataset from 2010-2020, train model with data from 2010- 2018, and test on data from 2019-2020): + 2 - Held-out validation cohort (e.g., cross-validation, leave-one-out cross validation, external validation): + 2   Method of evaluating model generalizability, select one of the following   - Internal validation (i.e., separate cohort not used for model training from the same institution): + 1 - Prospective validation: + 2 - External validation (i.e., separate cohort not used for model training from a different institution): + 3   Risk of data leakage, select one of the following   - Yes (i.e., data preprocessing, imputation, and/or dimension reduction performed prior to data splitting): 0 for entire item - No (i.e., held-out testing cohort): + 2 | /7 |
| 9 | **Sample size calculation**  Provide rationale for sample size required for model development (e.g., based on power calculation) | - If not reported: 0 for entire item - Sample size reported: + 2 - Number of events reported: + 2 - Details provided for sample size calculation (can be in supplementary material): + 1 | /5 |

| **Item** | **Description** | **Score** | **Total** |
| --- | --- | --- | --- |
| 10 | **Baseline**  Describe the baseline model that will serve as a comparison for the AI model(s) | - Existing model from prior literature: + 2 - Regression model using same features in AI model: + 2 - Domain expert (e.g., clinician judgement) or comparison to current standard of care (gold standard): + 4 | /8 |
| 11 | **Model description**  Describe the AI model(s) and software libraries investigated | - AI model(s): + 1 - Software libraries: + 1 | /2 |
| 12 | **Hyperparameter tuning**  Specify all model hyperparameters that were optimized, the search space for hyperparameter tuning, and evaluation metric(s) used to optimize parameters. (Details can be included in Supplementary Material) | - Hyperparameters that are tuned are listed: + 1 - Hyperparameter search strategy is described (e.g., random-, grid-search, etc.): + 1 - Optimization metric is specified (e.g., accuracy, AUROC, etc.): + 1   Search space for hyperparameters, select one of the following   - Not reported: + 0 - Reported for some of the listed hyperparameters, while others are missing or unclear:   + 1   - Reported for all listed hyperparameters, or reported for some and explicitly states that the others were set to their default values: + 2 | /5 |
| **Results** | | | |
| 13 | **Cohort characteristics**  Provide the total cohort size and summary statistics of the training, validation (if used), and testing cohorts, including incidence of the ground truth of interest | - Total cohort size, number of samples with missing data, and follow-up time (if applicable): + 1 - Summary statistics of each cohort to show similarities and differences among cohorts:   + 2   - Incidence of ground truth(s) of interest: + 1 | /4 |
| 14 | **Model specification**  Present the final AI model and specify the final panel of features included  and hyperparameters tuned. (Final hyperparameters can be listed in Supplementary Material) | - AI model reported: + 1 - Final set of features reported: + 1 - Final set of hyperparameters reported: + 1 | /3 |
| 15 | **Model evaluation**  List the evaluation metrics used to assess performance and calibration, including the justification for selection | Discrimination (e.g., AUROC, AUPRC, c-index, etc.), select one of the following   - No assessment of discrimination: + 0 - Some assessment of discrimination without statistical significance: + 1 - Some assessment of discrimination with statistical significance included (e.g., confidence intervals, p-values): + 2   Justification for discrimination metric, select one of the following   - No justification provided: + 0 - Rationale provided for which metric is most clinically relevant for the problem at hand: + 1   Calibration (e.g., calibration plots, calibration slope and intercept), select one of the following   - No assessment of calibration: + 0 - Statistical summary of calibration only (e.g., Hosmer-Lemeshow test, which does not indicate direction or magnitude of miscalibration): + 1 - Calibration plot reported: + 2 | /5 |
| 16 | **Clinical utility assessment**  Describe appropriate metrics for readers to understand the risk/benefit trade- offs of using the AI model at the specified decision threshold (e.g., decision curve analysis) | Select one of the following   - No assessment of clinical utility: + 0 - Sensitivity and specificity reported for a specified threshold: + 2 - Decision curve analysis or impact on clinical outcomes (e.g., overall survival, length of stay, readmission rates): + 5 | /5 |

| **Item** | **Description** | **Score** | **Total** |
| --- | --- | --- | --- |
| 17 | **Bias assessment**  Compare evaluation metrics for the AI model(s) and reference standard when stratified by patient- and task-specific subgroups to identify subgroups that benefit, are not helped at all, or harmed by the models. Patient-specific subgroups may include age group, gender, ethnicity, or socioeconomic status. Task-specific subgroups are disease-specific and may include risk stratification (e.g., low-, intermediate-, and high-risk disease in prostate cancer), or subtyping (e.g., different bacteria in positive blood cultures). | Patient-specific subgroup analysis   - Model performance (e.g., AUROC) evaluated across subgroup: + 1 - Clinical utility (e.g., decision curve analysis) evaluated across subgroup: + 1 - More than one subgroup evaluated for any of the above: + 1   Task-specific subgroup analysis   - Model performance (e.g., AUROC) evaluated across subgroup: + 1 - Clinical utility (e.g., decision curve analysis) evaluated across subgroup: + 1 - More than one subgroup evaluated for any of the above: + 1 | /6 |
| 18 | **Error analysis**  Analyze predictive errors to identify characteristics that are more prone to inaccurate predictions. Determine if there are any surprise errors (e.g., clearly inaccurate predictions based on clinical judgement). | - Analysis of predictive errors: + 2 - Assessment of surprise errors: + 2 | /4 |
| 19 | **Model explanation (optional)**  Describe methods used to explain AI models | Optional |  |
| **Discussion** | | | |
| 20 | **Critical analysis**  Describe the main findings of the study, including:   - New predictors of the ground truth of interest identified using AI - Strengths of the AI model(s) compared to the current models in the literature - Why the AI model(s) performed better/worse than what is currently available - (Optional) If feature importance rankings were used, describe whether they were aligned with clinical intuition and known prognostic factors | + 5 | /5 |
| 21 | **Implementation into clinical practice**  Describe how the AI model(s) can be applied to clinical practice, with respect to the potential to improve patient care, clinical decision-making, and/or efficiency | + 1 | /1 |
| 22 | **Limitations**  Discuss the limitations of the AI model(s), with consideration of the data, features, model(s), and/or biases | + 2 | /2 |
| **Other information** | | | |
| 23 | **Disclosures**  Disclose all financial relationships, sources of funding, and potential conflicts of interest | + 1 | /1 |
| 24 | **Transparency**  Share the data, source code, or release an application that runs the code. Data dictionary involves providing descriptions of all features and ground truth, with consideration of the following:   - Data type (i.e., categorical or numerical) - Method of collection or measurement (e.g., serum hemoglobin in g/dL) - Range of values (e.g., yes or no) | Data dictionary   - Description of all variables and how they were measured: + 1   Data availability, select one of the following   - Data available on request: + 1 - Data available on established data sharing repository: + 4   Model availability   - Nomogram/scoring system/website available to use model: + 1 - Trained model available: + 1 - Complete source code available: + 1 - Executable end-to-end (e.g., dependency file, documentation on how to run the code) available: + 2 | /10 |

| **Item** | **Description** | **Score** | **Total** |
| --- | --- | --- | --- |
| **Overall score: /100** | | | |

**eTable 2.** Scoring Rubric for Expert Ratings for Each Included Article

| **Criteria** | **1 (very weak)** | **2** | **3 (acceptable)** | **4** | **5 (very strong)** |
| --- | --- | --- | --- | --- | --- |
| **Feasibility of development** | Not feasible |  | May be feasible in select cases |  | Easily deployed |
| **Data collection** | Poor quality, limited validity |  |  |  | High quality |
| **Data preparation** | No description of data at all |  | Some key elements missing |  | Extremely thorough description |
| **Methods are implemented correctly** | Poor or incorrect implementation |  |  |  | Best practice in implementation applied |
| **Results are generalizable** | Unlikely to be a real result |  |  |  | Results are broadly generalizable |
| **Interpretation of findings** | Outlandish |  |  |  | Well-described, appropriate within context of results |
| **Model/code is available** | Not available |  | Upon reasonable request |  | Publicly available code repository |
| **Study is replicable** | Unlikely to be replicable |  |  |  | Very likely to be replicable |
| **Total /40** | | | | | |

**eTable 3.** APPRAISE-AI Tool on a High-Quality Image Analysis Study

The individual item scores and their corresponding explanations are provided.

**Article title** Validation and algorithmic audit of a deep learning system for the detection of proximal femoral fractures in patients in the emergency department: a diagnostic accuracy study

**DOI link** https://doi.org/10.1016/S2589-7500(22)00004-8

**Score Explanation [page number of study]**

| **Title** | | | |
| --- | --- | --- | --- |
| **1** | **Identify the report as an AI application to a specific clinical question. [Max score 1]** | **1** |  |
| i | The words artificial intelligence, AI, machine learning, deep learning, or other terminology related to artificial intelligence are reported in the title | Y | The study title indicates that a deep learning system is investigated [page 1]. |
| ii | The outcome of interest predicted by the AI model is reported in the title | Y | The study title indicates that the outcome of interest is the presence of a proximal femoral fracture [page 1]. |
| iii | The target population in which the AI model will be used is reported in the title | Y | The study title indicates that the target population involves patients in the emergency department [page 1]. |
| **Introduction** | | | |
| **2** | **Background: Describe the clinical problem and rationale for developing AI models. Review existing relevant literature exploring AI models for the problem being addressed. [Max score 1]** | **1** |  |
| i | The clinical context and rationale for developing/updating an AI model(s) to address the clinical problem are presented | Y | The authors describe how proximal femoral fractures are a common cause of hospitalization in elderly patients with a significant risk of morbidity and mortality. However, the current diagnostic pathway may  miss occult fractures and lead to increased costs. The authors propose that use of a deep learning model may improve diagnostic accuracy of proximal femoral fractures at first presentation [pages 1-2]. |
| ii | A synthesis of existing AI models that predict the same outcome is provided. If there are no existing models, this should be stated | Y | In the research in context, the authors highlight that only six studies utilized deep learning models for hip  fracture detection prior to their study. They summarize limitations of the existing literature, including limited external validation and the lack of algorithmic audits [page 2]. |
| **3** | **Objective and Problem: Clearly state what the proposed AI model(s) aims to address with respect to the study population and outcome. [Max score 1]** | **1** |  |
| i | The objectives are presented | Y | The authors outline the study objectives including 1) comparing their deep learning model against clinical experts, 2) external validation of their model on international data, and 3) performing an algorithmic audit to identify aberrant behaviour of their model [page 2]. |
| ii | The target population and outcome of interest are stated | Y | The authors specify that their previously developed deep learning model was developed to detect proximal femoral fractures (outcome of interest) based on initial x-rays in the emergency department (target  population) [page 1]. |
| **Methods** | | | |
| **4** | **Source of Data: Describe how the dataset was obtained (e.g., single/multi-center, local/national database, etc.), and study period. If relevant, the diversity of the dataset is also described (e.g., inclusion of community hospitals, low/middle income populations, and**  **institutions from other countries). [Max score 8]** | **4** |  |
| i | How many institutions were included in the dataset? | Multiple institutions | Two institutions were included in this study including the Royal Adelaide Hospital (Adelaide, SA, Australia) and Stanford University Medical Center (Stanford, CA, United States) [pages 2-3]. |
| ii | Was the study period (start and end dates) reported? | Y | The Royal Adelaide Hospital dataset included all frontal pelvic x-rays ordered from 01-Jan-2005 to 31-Dec- 2015. The Stanford University Medical Center dataset included lower extremity x-rays from 01-Jan-2003 to  31-Dec-2014 [pages 2-3]. |
| iii | Was the length of follow-up reported, if applicable? | Y | Length of follow-up was at least 6 months. This follow-up period was used to determine the ground truth (i.e., presence of proximal femoral fracture) [page 3]. |
| iv | What was the setting(s) of the institutions included in the data or inferred based on their description?  *If not reported or unknown, select No.*  Academic institutions | Y | The Royal Adelaide Hospital and Stanford University Medical Centers are both academic teaching hospitals [pages 2-3]. |
|  | Institutions from multiple (> 1) countries | Y | Australia and United States [pages 2-3] |
|  | Community-based or rural hospital(s) | N | Not specified in the study |

|  | Low/middle income patient populations | N | Not specified in the study |
| --- | --- | --- | --- |
| **5** | **Eligibility criteria: Specify all criteria for inclusion/exclusion of patients and features. Provide appropriate details (e.g., adults, age >**  **18) and rationale. [Max score 3]** | **3** |  |
| i | Inclusion criteria are provided | Y | For the Royal Adelaide Hospital dataset, all frontal pelvic x-rays were included, regardless of x-ray equipment and imaging parameters. For the Stanford University Medical Center dataset, all lower extremity  x-rays were included, of which a random selection of 46 fracture and 100 non-fracture cases were selected [pages 2-3]. |
| ii | Exclusion criteria are provided | Y | For the Royal Adelaide Hospital dataset, cases were excluded if surgical hardware is seen in the x-ray or if there were no frontal pelvic x-rays available. For the Stanford University Medical Center dataset, cases  were excluded if surgical hardware is seen in the x-ray or if personal health information is included in the raw image [pages 2-3]. |
| iii | Details and rationale for criteria are provided | Y | The authors explained that cases with surgical hardware were excluded since they represent a different class  of hip injury, while the target population for their deep learning model is focused on preoperative patients. X-rays with embedded personal health information were excluded for privacy reasons [pages 2-3]. |
| **6** | **Ground truth: Define the ground truth of interest. Describe how it was collected (e.g., manual annotation by experts) and encoded (e.g.,**  **binary, categorical, dichotomized continuous, continuous variable, etc.). [Max score 6]** | **6** |  |
| i | Ground truth of interest is clearly defined  *For unsupervised learning, describe what measure(s) and associated data will be used to assess cluster validity (e.g., correlating disease- specific features with overall survival)* | Y | Presence of a proximal femoral fracture [page 3] |
| ii | How was the ground truth determined? | Multiple (>1), experts | Ground truth was determined through combination of multiple means including 1) x-ray reports, 2) follow- up imaging such as x-rays, CT, or MRI, and 3) operative reports. Since not all patients were surgically validated (i.e., underwent surgery to treat their fracture), this was not scored as “Objective, well-captured ground truth”. Given the multiple assessments by clinical experts, this was scored as “Multiple (>1),  experts” [page 3]. |
| **7** | **Data abstraction, cleaning, preparation: Describe the methods used to develop the final dataset, with consideration of feature abstraction, handling of missing data, feature engineering, and removal of**  **features. [Max score 7]** | **7** |  |
| i | Rationale provided for choice of candidate features (e.g., based on prior research, clinical relevance, available data, etc.) | Y | Plain frontal pelvic radiographs were used since this is most commonly ordered at the time of initial presentation in emergency department [page 2]. |
| ii | Time-windows for abstracted features are specified (e.g., vital signs recorded within the past 12 hours will be used to predict sepsis) | Y | All included x-rays were from preoperative hips at initial presentation [pages 2-3]. |
| iii | How was missing data handled?  *If there is no missing data, it should be clearly stated that there is no*  *missing data, select Not applicable. If it is unclear whether there is missing data or how it was handled, select Not reported* | Not applicable | This is not applicable since the deep learning model requires a frontal pelvic x-ray image to make a prediction. Patients without frontal pelvic x-rays were excluded and this is indicated in Figure 1 [page 4]. |
| iv | Transformation/Augmentation: Details provided for how data was altered to change its representation (e.g., normalization, log-transformation, one- hot encoding, image rotation, image translation, adjusting image contrast) *If not performed, it should be clearly stated that it was not performed, select Not applicable. If it is unclear whether it was performed or not*  *explicitly stated, select No* | Y | Transformation and augmentation procedures included standardizing pixel intensities, image translation, rotations, shears, and histogram matching [appendix page 8, 10]. |
|  | Modification/Cleaning: Details provided for how data was altered in a non-uniform manner (e.g., outlier removal).  *If not performed, it should be clearly stated that it was not performed, select Not applicable. If it is unclear whether it was performed or not*  *explicitly stated, select No* | Y | Bounding boxes were created to localize and separate the left and right hips [appendix page 9]. |
| v | Outline any methods used to remove features (e.g., clinical judgement, principal component analysis, recursive feature elimination, correlation, or ablation analysis), if applicable  *If not performed, it should be clearly stated that it was not performed,* | Not applicable | Not performed |

|  | *select Not applicable. If it is unclear whether it was performed or not explicitly stated, select No.* |  |  |
| --- | --- | --- | --- |
| **8** | **Data splitting: Specify how the data was divided into the training, validation, and testing cohorts. [Max score 7]** | **7** |  |
| i | What was the method of data splitting used? | Held-out validation cohort (e.g., cross- validation, leave-one-out cross validation, external  validation) | Both random split at the patient-level (Royal Adelaide Hospital dataset) and external validation (Stanford University Medical Center dataset) were used. The option that yielded the higher possible score (external validation) was selected [pages 2-3]. |
| ii | What was the method used to evaluate model generalizability | External validation (i.e, separate cohort not used for model training from a  different institution) | See explanation for item 8i [pages 2-3] |
| iii | Were there any concerns of data leakage (i.e., data preprocessing performed prior to data splitting, training and testing on the same data)? | N | No concerns for data leakage. Pixel intensities were standardized to within 0 to 4095 [appendix pages 7-9]. |
| **9** | **Sample size calculation: Provide rationale for sample size required for model development (e.g., based on power calculation). [Max score**  **5]** | **0** |  |
| i | Minimum sample size required reported | N | Not specified. However, the authors state that the number of cases included in the multi-reader, multi-case study maximized the sample size while balancing what can be reasonably expected from clinicians. They  compared their sample size against similar studies in the discussion [pages 3, 7]. |
| ii | Minimum number of events required reported | N | Not specified |
| iii | Details provided for sample size calculation (e.g. assumptions for event rates, target performance, power, significance level). Can be provided in  supplementary material | N | Not specified |
| **10** | **Baseline: Describe the baseline model that will serve as a comparison for the AI model(s). [Max score 8]** | **4** |  |
| i | Existing model from prior literature used for comparison | N | Not included |
| ii | Regression model using same features in AI model used for comparison | N | Not included and not possible for this image analysis study |
| iii | Domain expert (e.g., clinician judgement) or current standard of care (gold standard) used for comparison | Y | The deep learning model was compared against the current gold standard – interpretation by a radiologist. Five radiologists (three musculoskeletal specialists and two general radiologists) with 5 to 19 years of  clinical experience post-fellowship were included [page 3]. |
| **11** | **Model description: Describe the AI model(s) and software libraries investigated. [Max score 2]** | **2** |  |
| i | Type of AI model(s) reported (e.g., random forest, support vector machine, convolutional neural network) | Y | DenseNet with 172 layers, 12 features/units per layer, and 1,434,176 parameters [page 2, appendix pages 9-  10] |
| ii | Software libraries reported (e.g., scikit-learn 1.1.2) | Y | PyTorch [appendix page 10] |
| **12** | **Hyperparameter tuning: Specify all model hyperparameters that were optimized, the search space for hyperparameter tuning, and evaluation metric(s) used to optimize parameters. Details can be included in Supplementary Material. [Max score 5]** | **3** |  |
| i | Hyperparameters that are tuned are listed (e.g., number of trees, max depth, number of neurons) | Y | Layer width, choice of activation function and leak rate, use of secondary loss function, types and extent of data augmentation, level of regularisation, and learning rate [appendix page 9]. |
| ii | Optimization metric is specified (e.g., accuracy, AUROC, etc.) | Y | Two loss functions were optimized: 1) primary loss from the presence/absence of proximal femoral fracture and 2) secondary loss on specific fracture location (intra-capsular, extra-capsular, no fracture) [appendix  page 10]. |
| iii | Hyperparameter search strategy is described (e.g., random-, grid-search, etc.) | Y | Grid search [appendix page 9] |
| iv | Search space for hyperparameters are provided | Not reported | Not specified |
| **Results** | | | |
| **13** | **Cohort characteristics: Provide the total cohort size and summary**  **statistics of the training, validation (if used), and testing cohorts, including incidence of the ground truth of interest. [Max score 4]** | **4** |  |
| i | Total cohort size, number of samples with missing data, and follow-up time (if applicable) are reported | Y | Table 1 [page 5] |

| ii | Summary statistics of each cohort provided to show similarities and differences among cohorts | Y | Table 1 [page 5] |
| --- | --- | --- | --- |
| iii | Incidence of ground truth(s) of interest is reported | Y | Table 1 [page 5] |
| **14** | **Model specification: Present the final AI model(s) and specify the final panel of features included and hyperparameters tuned. Final hyperparameters can be listed in Supplementary Material. [Max**  **score 3]** | **3** |  |
| i | Type of AI model(s) is reported | Y | Model was previously developed and specified in both the Methods and Appendix [page 2, appendix pages 9-10]. |
| ii | Final set of features are reported | Y | Frontal pelvic x-ray images converted into numpy arrays [appendix page 8]. |
| iii | Final set of hyperparameters are reported | Y | The final convolutional neural network included 172 layers and 12 features/units per layer. It used leaky RELU non-linear activations with a leak rate of 0.5 and pre-activation batch normalisation. Dropout rate  was 0.2 with a weight decay rate of 1e-5. The network was trained for 25 epochs via stochastic gradient descent using the Adam optimiser, learning rate of 0.0001, and batch size of 14 [appendix page 10]. |
| **15** | **Model evaluation: List the evaluation metrics used to assess**  **performance and calibration, including the justification for selection. [Max score 5]** | **3** |  |
| i | Measure(s) for model discrimination is reported (e.g., AUROC, AUPRC, c-index, etc.)  *If multiple measures of discrimination are provided and at least one includes a measure of statistical significance, select Measure(s) with statistical significance* | Measure(s) with statistical significance (e.g., confidence interval, standard error,  p-value) | Area under the receiver operating characteristic curve with 95% confidence intervals determined using 10,000 bootstrap samples [pages 3-4]. |
| ii | Rationale provided for which metric is most clinically relevant for the problem at hand | Y | Metric is appropriate for binary outcome (fracture vs no fracture) [page 3]. |
| iii | Measure(s) for model calibration is reported (e.g., calibration plots, calibration slope and intercept)  *If both calibration plot and statistical summary of calibration are provided, select Calibration plot* | Not reported | Not performed |
| **16** | **Clinical utility assessment: Describe appropriate metrics for readers to understand the risk/benefit trade-offs of using the AI model at the specified decision threshold (e.g., decision curve analysis). [Max score 5]** | **2** |  |
| i | Measure(s) of clinical utility is reported  *If both sensitivity or specificity for a specified threshold and decision curve analysis are provided, select Decision curve analysis* | Sensitivity or specificity reported for a specified threshold | Sensitivity and specificity of the deep learning model are provided for several operating points [page 5]. |
| **17** | **Bias assessment: Compare evaluation metrics for the AI model(s) and reference standard when stratified by patient- and task-specific subgroups to identify subgroups that benefit, are not helped at all, or harmed by the models.**  **Patient-specific subgroups may include age group, gender, ethnicity, or socioeconomic status.**  **Task-specific subgroups are disease-specific and may include risk stratification (e.g., low-, intermediate-, and high-risk disease in prostate cancer), or subtyping (e.g., different bacteria in positive**  **blood cultures). [Max score 6]** | **4** |  |
| i | Patient-specific: Performance (e.g., AUROC) is evaluated across at least one subgroup | Y | Sex (male, female), age (< 40, 40-60, 61-80, > 80 years) [page 6]. |
| ii | Patient-specific: Clinical utility (e.g., sensitivity or specificity for a specified threshold) is evaluated across at least one subgroup | N | Not performed |
| iii | Patient-specific: More than one subgroup is evaluated in either performance or clinical utility | Y | See explanation for item 17i [page 6]. |
| iv | Task-specific: Performance (e.g., AUROC) is evaluated across at least one subgroup | Y | Type of fracture (subtle, mild, moderate, severe displacement, comminuted), location of fracture (subcapital, cervical, pertrochanteric, subtrochanteric) [page 6]. |
| v | Task-specific: Clinical utility (e.g., sensitivity or specificity for a specified threshold) is evaluated across at least one subgroup | N | Not performed |

| vi | Task-specific: More than one subgroup is evaluated in either model performance or clinical utility | Y | See explanation for item 17iv [page 6]. |
| --- | --- | --- | --- |
| **18** | **Error analysis: Analyze predictive errors to identify characteristics that are more prone to inaccurate predictions. Determine if there are**  **any surprise errors (e.g., clearly inaccurate predictions based on clinical judgement). [Max score 4]** | **4** |  |
| i | Analysis of predictive errors is reported | Y | Overrepresented errors included non-displaced fractures and cases with abnormal bones or joints [appendix page 12] |
| ii | Analysis of surprise errors is reported | Y | Two surprise false negatives included 1) a minimally displaced subtrochanteric fracture in a patient with Paget’s disease and 2) a heavily displaced subtrochanteric fracture with the fracture elements forming a  pseudo-Shenton’s line. The one false positive was a case with a severely deformed femoral head, suspected due to a childhood injury but has not progressed due to osteoarthritis [appendix pages 12-14]. |
| **19** | **Model explanation: Describe methods used to explain AI models (e.g., SHAP, LIME, Grad-CAM) [Not scored]** | **0** |  |
| i | Model explanations are provided | Y | Grad-CAM saliency maps [appendix pages 13-14] |
| **Discussion** | | | |
| **20** | **Critical analysis: Describe main findings and limitations of the study. [Max score 5]** | **5** |  |
| i | An overall interpretation of the results is presented, which may include:   - New predictors of the ground truth of interest discovered using AI - Strengths of the AI model(s) compared to current models in the literature - Why the AI model(s) performed better/worse than what is currently available? - (Optional) If feature importance rankings were used, describe whether they were aligned with clinical intuition and known prognostic factors | Y | The authors highlight that their deep learning model was generalizable to an international cohort. It outperformed radiologists and the reported performance of a previously developed AI model. They mention the significant drop in sensitivity using the pre-specified operating point, which would limit its clinical utility. They summarize the key errors identified in their algorithmic audit [pages 6-7] |
| **21** | **Implementation into clinical practice: Describe how the AI model(s) can be applied to clinical practice, with respect to the potential to**  **improve patient care, clinical decision-making, and/or efficiency. [Max score 1]** | **1** |  |
| i | Potential application(s) to clinical practice and future directions are discussed | Y | The authors outline how their deep learning model can be implemented into clinical workflows and  mitigation strategies to address limitations outlined in their algorithmic audit [page 7, appendix pages 16- 20]. |
| **22** | **Limitations: Discuss the limitations of the AI model(s), with consideration of the data, features, model(s), and/or biases. [Max score 2]** | **2** |  |
| i | Limitations are discussed | Y | The authors outline limitations including 1) exclusion of patients with surgical hardware, 2) low sample size of the multi-reader, multi-case study, 3) lack of racial or ethnicity information for patient-specific subgroup testing, and 4) findings from the algorithmic audit may not be statistically reliable [page 7]. |
| **Other Information** | | | |
| **23** | **Disclosures: Disclose all financial relationships, sources of funding, and potential conflicts of interest. [Max score 1]** | **1** |  |
| i | All relevant disclosures are reported | Y | No funding source [page 5] |
| **24** | **Transparency: Share the data, data dictionary, source code, or release an application that runs the code. [Max score 10]** | **2** |  |
| i | Data dictionary: A description is provided for all features and ground truth, with consideration of the following:   - Data type (i.e., categorical or numerical) - Method of collection or measurement (e.g., serum hemoglobin in g/dL) - Range of values (e.g., yes or no, 0.5-250 g/dL) | N | Descriptions of some, but not all, of the data are provided such as fracture location and character [page 4]. |
| ii | Data availability: How can other researchers access the data used in the study?  *Data availability needs to be explicitly stated to receive points* | Available on request | The data sharing statement indicates that the derived data is available upon requests to the corresponding author [page 8]. |
| iii | Model availability: How can other researchers access the model(s) used in the study? | N | Not provided |

|  | Nomogram/scoring system/website available to use model for single predictions |  |  |
| --- | --- | --- | --- |
|  | Trained model available to generate prediction in bulk (i.e., from a dataset) | N | Not provided. Data sharing statement indicates that the model is available upon requests to the corresponding author [page 8]. |
|  | Complete source code available | N | Not provided |
|  | Executable end-to-end (e.g., dependency file, documentation on how to run the code) available | N | Not provided |
|  | | | |
|  | **Overall APPRAISE-AI score (out of 100)** | **69** | |
|  | **Quality based on overall APPRAISE-AI score** | **High** | |
|  | **Clinical Relevance (out of 4)** | **4** | |
|  | **Data Quality (out of 24)** | **20** | |
|  | **Methodological Conduct (out of 20)** | **11** | |
|  | **Robustness of Results (out of 20)** | **13** | |
|  | **Reporting Quality (out of 12)** | **12** | |
|  | **Reproducibility (out of 20)** | **9** | |

**eTable 4.** APPRAISE-AI Tool on a High-Quality Classification Study

The individual item scores and their corresponding explanations are provided.

**Article title** Long-term mortality risk stratification of liver transplant recipients: real-time application of deep learning algorithms on longitudinal data

**DOI link** https://doi.org/10.1016/S2589-7500(21)00040-6

**Score Explanation [page number of study]**

| **Title** | | | |
| --- | --- | --- | --- |
| **1** | **Identify the report as an AI application to a specific clinical question. [Max score 1]** | **1** |  |
| i | The words artificial intelligence, AI, machine learning, deep learning, or other terminology related to artificial intelligence are reported in the title | Y | The study title indicates that a deep learning system is investigated [page 1]. |
| ii | The outcome of interest predicted by the AI model is reported in the title | Y | The study title indicates that the outcome of interest is mortality [page 1]. |
| iii | The target population in which the AI model will be used is reported in the title | Y | The study title indicates that the target population involves liver transplant recipients [page 1]. |
| **Introduction** | | | |
| **2** | **Background: Describe the clinical problem and rationale for developing AI models. Review existing relevant literature exploring AI models for the problem being addressed. [Max score 1]** | **1** |  |
| i | The clinical context and rationale for developing/updating an AI model(s) to address the clinical problem are presented | Y | The authors describe how long-term life expectancy following liver transplantation may be impacted by graft failure, infections, cardiovascular complications, and cancer. While several risk factors for these long- term complications have been identified, they have not been integrated in a comprehensive and longitudinal manner, which is possible due to the longitudinal follow-up that is standard of care in this patient population. The authors propose the use of a deep learning model utilizing longitudinal data to provide more  accurate prognostication of mortality due to graft failure, infection, cancer, or cardiovascular causes [pages 1-2]. |
| ii | A synthesis of existing AI models that predict the same outcome is provided. If there are no existing models, this should be stated | Y | In the research in context, the authors found that no studies have investigated the use of longitudinal data to predict liver transplant outcomes [page 2]. |
| **3** | **Objective and Problem: Clearly state what the proposed AI model(s) aims to address with respect to the study population and outcome.**  **[Max score 1]** | **1** |  |
| i | The objectives are presented | Y | The authors outline the study objectives which include developing and validating a deep learning model to predict post-liver transplant mortality at 1 and 5 years after each clinic visit due to graft failure, infection,  cancer, or cardiovascular causes [page 2]. |
| ii | The target population and outcome of interest are stated | Y | The target population is liver transplant recipients. The outcome of interest is 1- and 5-year mortality due to graft failure, infection, cancer, or cardiovascular causes [page 2]. |
| **Methods** | | | |
| **4** | **Source of Data: Describe how the dataset was obtained (e.g., single/multi-center, local/national database, etc.), and study period. If relevant, the diversity of the dataset is also described (e.g., inclusion of community hospitals, low/middle income populations, and**  **institutions from other countries). [Max score 8]** | **4** |  |
| i | How many institutions were included in the dataset? | Multiple institutions | Two institutions were included in this study including the University Health Network (UHN, Toronto, Canada) and the Scientific Registry of Transplant Recipients (SRTR, a national registry of transplant  patients in the United States) [page 2]. |
| ii | Was the study period (start and end dates) reported? | Y | The SRTR dataset included all liver transplant recipients from 01-Jan-2003 to 30-Sep-2014. The UHN dataset included all liver transplant recipients from 01-Dec-1986 to 30-Sep-2014 [page 3]. |
| iii | Was the length of follow-up reported, if applicable? | Y | Length of follow-up was at least 5 years following liver transplantation. This follow-up period was used to assess 1- and 5-year mortality [page 3]. |
| iv | What was the setting(s) of the institutions included in the data or inferred based on their description?  *If not reported or unknown, select No.*  Academic institutions | Y | Liver transplantation is typically performed only at academic institutions. UHN is an academic teaching hospital [pages 2-3]. |
|  | Institutions from multiple (> 1) countries | Y | Canada and United States [page 2] |

|  | Community-based or rural hospital(s) | N | Not specified in the study |
| --- | --- | --- | --- |
|  | Low/middle income patient populations | N | Not specified in the study |
| **5** | **Eligibility criteria: Specify all criteria for inclusion/exclusion of**  **patients and features. Provide appropriate details (e.g., adults, age > 18) and rationale. [Max score 3]** | **3** |  |
| i | Inclusion criteria are provided | Y | All liver transplant recipients in the specified study period (item 4ii) were included [page 2]. |
| ii | Exclusion criteria are provided | Y | Patients were excluded if 1) the cause of death was outside the four categories of interest (graft failure,  infection, cancer, or cardiovascular cause), 2) age < 18, 3) patients who survived but had < 5 years of follow-up, 4) multi-organ transplant recipients or 5) missing lab values in the UHN dataset [pages 2-3]. |
| iii | Details and rationale for criteria are provided | Y | Patients with less than 5 years of follow-up were excluded since they could not be used to determine 5-year mortality. Multi-organ recipients were excluded due to small sample size and likely different dynamics  compared to single-organ recipients. UHN patients with missing lab values were excluded due to the importance of these features and the large number of missing values for these patients [pages 2-3]. |
| **6** | **Ground truth: Define the ground truth of interest. Describe how it was collected (e.g., manual annotation by experts) and encoded (e.g., binary, categorical, dichotomized continuous, continuous variable,**  **etc.). [Max score 6]** | **6** |  |
| i | Ground truth of interest is clearly defined  *For unsupervised learning, describe what measure(s) and associated data will be used to assess cluster validity (e.g., correlating disease- specific features with overall survival)* | Y | Death within 1 or 5 years due to graft failure, infection, cancer, or cardiovascular cause [page 3] |
| ii | How was the ground truth determined? | Multiple (>1), experts | Ground truth was determined using International Classification of Diseases codes for the SRTR dataset, and manual chart review for the UHN dataset. Since not all outcomes were determined via diagnostic codes, this was not scored as “Objective, well-captured ground truth”. Given the multiple assessments by clinical  experts through chart review, this was scored as “Multiple (>1), experts” [page 3]. |
| **7** | **Data abstraction, cleaning, preparation: Describe the methods used to develop the final dataset, with consideration of feature abstraction, handling of missing data, feature engineering, and removal of**  **features. [Max score 7]** | **3** |  |
| i | Rationale provided for choice of candidate features (e.g., based on prior research, clinical relevance, available data, etc.) | Y | Features were selected based on availability and known risk factors of complications post-transplantation [pages 1, 4]. |
| ii | Time-windows for abstracted features are specified (e.g., vital signs recorded within the past 12 hours will be used to predict sepsis) | N | Not specified |
| iii | How was missing data handled?  *If there is no missing data, it should be clearly stated that there is no missing data, select Not applicable. If it is unclear whether there is missing data or how it was handled, select Not reported* | Explicit modeling of missing data with appropriate justification (e.g., directly through AI model, multiple imputation, or other  statistical approaches) | Imputation was primarily done through forward-filling. The authors also experimented with median- and mean-filling, and random drawing from the training distribution. They found that the forward-filling approach yielded the best AUROC [page 4, appendix pages 3, 19]. |
| iv | Transformation/Augmentation: Details provided for how data was altered to change its representation (e.g., normalization, log-transformation, one- hot encoding, image rotation, image translation, adjusting image contrast) *If not performed, it should be clearly stated that it was not performed, select Not applicable. If it is unclear whether it was performed or not*  *explicitly stated, select No* | N | Not specified |
|  | Modification/Cleaning: Details provided for how data was altered in a non-uniform manner (e.g., outlier removal).  *If not performed, it should be clearly stated that it was not performed,*  *select Not applicable. If it is unclear whether it was performed or not explicitly stated, select No* | N | Not specified |
| v | Outline any methods used to remove features (e.g., clinical judgement, principal component analysis, recursive feature elimination, correlation, or ablation analysis), if applicable  *If not performed, it should be clearly stated that it was not performed,* | N | Not specified |

|  | *select Not applicable. If it is unclear whether it was performed or not explicitly stated, select No.* |  |  |
| --- | --- | --- | --- |
| **8** | **Data splitting: Specify how the data was divided into the training, validation, and testing cohorts. [Max score 7]** | **5** |  |
| i | What was the method of data splitting used? | Held-out validation cohort (e.g., cross- validation, leave-one-out cross validation, external  validation) | A random 80:10:10 training/tuning/validation strategy was used for the SRTR dataset, while a 5-fold stratified cross validation strategy was used for the UHN dataset. As per Table 1, the option that yields the higher value (cross validation) was selected [page 4]. |
| ii | What was the method used to evaluate model generalizability | Internal validation (i.e., separate cohort not used for model training from  the same institution) | Although two institutions (SRTR and UHN) were included, they were both used in model development. Therefore, external validation was not selected [page 4] |
| iii | Were there any concerns of data leakage (i.e., data preprocessing performed prior to data splitting, training, and testing on the same data)? | N | No concerns for data leakage [pages 2-4]. |
| **9** | **Sample size calculation: Provide rationale for sample size required for model development (e.g., based on power calculation). [Max score**  **5]** | **0** |  |
| i | Minimum sample size required reported | N | Not specified |
| ii | Minimum number of events required reported | N | Not specified |
| iii | Details provided for sample size calculation (e.g. assumptions for event rates, target performance, power, significance level). Can be provided in supplementary material | N | Not specified |
| **10** | **Baseline: Describe the baseline model that will serve as a comparison for the AI model(s). [Max score 8]** | **2** |  |
| i | Existing model from prior literature used for comparison | N | Not included |
| ii | Regression model using same features in AI model used for comparison | Y | Logistic regression model [page 3] |
| iii | Domain expert (e.g., clinician judgement) or current standard of care (gold standard) used for comparison | N | Not included |
| **11** | **Model description: Describe the AI model(s) and software libraries investigated. [Max score 2]** | **2** |  |
| i | Type of AI model(s) reported (e.g., random forest, support vector  machine, convolutional neural network) | Y | Multilayer perceptron, recurrent neural network, temporal convolutional network, Transformer [page 3,  appendix pages 16-18] |
| ii | Software libraries reported (e.g., scikit-learn 1.1.2) | Y | PyTorch 1.1.0, sklearn [appendix pages 3-4] |
| **12** | **Hyperparameter tuning: Specify all model hyperparameters that were optimized, the search space for hyperparameter tuning, and evaluation metric(s) used to optimize parameters. Details can be included in Supplementary Material. [Max score 5]** | **5** |  |
| i | Hyperparameters that are tuned are listed (e.g., number of trees, max depth, number of neurons) | Y | Logistic regression: solver = lbfgs, regularization = 1  Hyperparameters used for the deep learning models are listed in Supplementary Table 1  Specific architecture choices for the deep learning models are listed in Supplementary Tables 2-4 [appendix pages 4-6]. |
| ii | Optimization metric is specified (e.g., accuracy, AUROC, etc.) | Y | AUROC [appendix page 4] |
| iii | Hyperparameter search strategy is described (e.g., random-, grid-search, etc.) | Y | Bayesian optimization framework using a tree-structured Parzen estimator [appendix page 4] |
| iv | Search space for hyperparameters are provided | Reported for all listed hyperparameters, or reported for some and explicitly states that the others were set to their  default values | Supplementary Table 1 [appendix page 5] |
| **Results** | | | |
| **13** | **Cohort characteristics: Provide the total cohort size and summary statistics of the training, validation (if used), and testing cohorts,**  **including incidence of the ground truth of interest. [Max score 4]** | **4** |  |

| i | Total cohort size, number of samples with missing data, and follow-up time (if applicable) are reported | Y | Figure 1 [page 3] |
| --- | --- | --- | --- |
| ii | Summary statistics of each cohort provided to show similarities and differences among cohorts | Y | Supplementary Table 8 [appendix page 5] |
| iii | Incidence of ground truth(s) of interest is reported | Y | Figure 1 [page 3] |
| **14** | **Model specification: Present the final AI model(s) and specify the final panel of features included and hyperparameters tuned. Final**  **hyperparameters can be listed in Supplementary Material. [Max score 3]** | **2** |  |
| i | Type of AI model(s) is reported | Y | Models were specified in both the Methods and Appendix [page 3, appendix pages 16-18]. |
| ii | Final set of features are reported | Y | Supplementary Tables 5-7 [appendix pages 6-11]. |
| iii | Final set of hyperparameters are reported | N | While the search space was provided, the final set of hyperparameters was not specified. |
| **15** | **Model evaluation: List the evaluation metrics used to assess performance and calibration, including the justification for selection.**  **[Max score 5]** | **3** |  |
| i | Measure(s) for model discrimination is reported (e.g., AUROC, AUPRC, c-index, etc.)  *If multiple measures of discrimination are provided and at least one includes a measure of statistical significance, select Measure(s) with statistical significance* | Measure(s) with statistical significance (e.g., confidence  interval, standard error, p-value) | Area under the receiver operating characteristic (AUROC) curve with 99% confidence intervals determined using 100 bootstrap samples, area under the precision-recall curve [page 4, appendix page 24]. |
| ii | Rationale provided for which metric is most clinically relevant for the problem at hand | Y | AUROC was described as the standard measure for machine learning classification problems [page 4]. |
| iii | Measure(s) for model calibration is reported (e.g., calibration plots, calibration slope and intercept)  *If both calibration plot and statistical summary of calibration are provided, select Calibration plot* | Not reported | Not performed |
| **16** | **Clinical utility assessment: Describe appropriate metrics for readers to understand the risk/benefit trade-offs of using the AI model at the specified decision threshold (e.g., decision curve analysis). [Max score 5]** | **2** |  |
| i | Measure(s) of clinical utility is reported  *If both sensitivity or specificity for a specified threshold and decision curve analysis are provided, select Decision curve analysis* | Sensitivity or specificity reported for a specified threshold | Sensitivity and specificity of the Transformer model provided in Supplementary Figure 8 [appendix page 23]. |
| **17** | **Bias assessment: Compare evaluation metrics for the AI model(s) and reference standard when stratified by patient- and task-specific subgroups to identify subgroups that benefit, are not helped at all, or harmed by the models.**  **Patient-specific subgroups may include age group, gender, ethnicity, or socioeconomic status.**  **Task-specific subgroups are disease-specific and may include risk stratification (e.g., low-, intermediate-, and high-risk disease in**  **prostate cancer), or subtyping (e.g., different bacteria in positive blood cultures). [Max score 6]** | **2** |  |
| i | Patient-specific: Performance (e.g., AUROC) is evaluated across at least one subgroup | Y | Years after transplantation (1 to 5, Figure 2D) [page 5] |
| ii | Patient-specific: Clinical utility (e.g., sensitivity or specificity for a specified threshold) is evaluated across at least one subgroup | N | Not performed |
| iii | Patient-specific: More than one subgroup is evaluated in either performance or clinical utility | N | Not performed |
| iv | Task-specific: Performance (e.g., AUROC) is evaluated across at least one subgroup | Y | Hepatitis C virus status (positive vs negative, Supplementary Table 10) [appendix page 14] |
| v | Task-specific: Clinical utility (e.g., sensitivity or specificity for a specified threshold) is evaluated across at least one subgroup | N | Not performed |
| vi | Task-specific: More than one subgroup is evaluated in either model performance or clinical utility | N | Not performed |

| **18** | **Error analysis: Analyze predictive errors to identify characteristics that are more prone to inaccurate predictions. Determine if there are any surprise errors (e.g., clearly inaccurate predictions based on**  **clinical judgement). [Max score 4]** | **0** |  |
| --- | --- | --- | --- |
| i | Analysis of predictive errors is reported | N | Not performed |
| ii | Analysis of surprise errors is reported | N | Not performed |
| **19** | **Model explanation: Describe methods used to explain AI models (e.g., SHAP, LIME, Grad-CAM) [Not scored]** | **0** |  |
| i | Model explanations are provided | Y | Saliency, SHAP, occlusion, Sobol (Supplementary Figures 5-6) [appendix page 20] |
| **Discussion** | | | |
| **20** | **Critical analysis: Describe main findings and limitations of the study. [Max score 5]** | **5** |  |
| i | An overall interpretation of the results is presented, which may include:   - New predictors of the ground truth of interest discovered using AI - Strengths of the AI model(s) compared to current models in the literature - Why the AI model(s) performed better/worse than what is currently available? - (Optional) If feature importance rankings were used, describe whether they were aligned with clinical intuition and known prognostic factors | Y | The authors highlight the strengths of their Transformer model including the integration of longitudinal data, prediction of major causes of death, and long-term mortality prediction. They reviewed the most important features for each of their outcomes, which aligned with the existing literature [pages 8-9]. |
| **21** | **Implementation into clinical practice: Describe how the AI model(s) can be applied to clinical practice, with respect to the potential to**  **improve patient care, clinical decision-making, and/or efficiency. [Max score 1]** | **1** |  |
| i | Potential application(s) to clinical practice and future directions are discussed | Y | The authors outline how their Transformer model can be implemented to predict 1- and 5-year mortality at each follow-up [page 9]. |
| **22** | **Limitations: Discuss the limitations of the AI model(s), with consideration of the data, features, model(s), and/or biases. [Max score 2]** | **2** |  |
| i | Limitations are discussed | Y | The authors outline limitations including 1) missing data and cause of death in the SRTR dataset, 2) inability to identify causal relationships, 3) lack of external validation, 4) lack of race and ethnicity information in the UHN dataset, 5) exclusion of other causes of death, and 6) shift in patient distribution  (overrepresentation of NAFLD patients, change in immunosuppression) [pages 9-10]. |
| **Other Information** | | | |
| **23** | **Disclosures: Disclose all financial relationships, sources of funding, and potential conflicts of interest. [Max score 1]** | **1** |  |
| i | All relevant disclosures are reported | Y | No funding source [page 4] |
| **24** | **Transparency: Share the data, data dictionary, source code, or release an application that runs the code. [Max score 10]** | **9** |  |
| i | Data dictionary: A description is provided for all features and ground truth, with consideration of the following:   - Data type (i.e., categorical or numerical) - Method of collection or measurement (e.g., serum hemoglobin in g/dL) - Range of values (e.g., yes or no, 0.5-250 g/dL) | Y | Supplementary Tables 5-7 [appendix pages 6-11]. |
| ii | Data availability: How can other researchers access the data used in the study?  *Data availability needs to be explicitly stated to receive points* | Available on an  established data sharing repository (e.g., MIMIC) | The data sharing statement indicates that the SRTR data is publicly available in the national registry,  however the UHN data is not available. As per Table 1, the option that yields the higher value (publicly available in established repository) was selected [page 10] |
| iii | Model availability: How can other researchers access the model(s) used in the study?  Nomogram/scoring system/website available to use model for single predictions | N | Not provided |
|  | Trained model available to generate prediction in bulk (i.e., from a dataset) | Y | Data sharing statement includes a Github repository to their models [page 10]. |
|  | Complete source code available | Y | Data sharing statement includes a Github repository to their models [page 10]. |

|  | Executable end-to-end (e.g., dependency file, documentation on how to run the code) available | Y | Data sharing statement includes a Github repository to their models with documentation on how to run their code [page 10]. |
| --- | --- | --- | --- |
|  | | | |
|  | **Overall APPRAISE-AI score (out of 100)** | **64** | |
|  | **Quality based on overall APPRAISE-AI score** | **High** | |
|  | **Clinical Relevance (out of 4)** | **4** | |
|  | **Data Quality (out of 24)** | **16** | |
|  | **Methodological Conduct (out of 20)** | **7** | |
|  | **Robustness of Results (out of 20)** | **7** | |
|  | **Reporting Quality (out of 12)** | **12** | |
|  | **Reproducibility (out of 20)** | **18** | |

**eTable 5.** APPRAISE-AI Tool on a High-Quality Survival Analysis Study The individual item scores and their corresponding explanations are provided.

**Article title** Application of a novel machine learning framework for predicting non-metastatic prostate cancer-specific mortality in men using the Surveillance, Epidemiology, and End Results (SEER) database

**DOI link** https://doi.org/10.1016/s2589-7500(20)30314-9

**Score Explanation [page number of study]**

| **Title** | | | |
| --- | --- | --- | --- |
| **1** | **Identify the report as an AI application to a specific clinical question. [Max score 1]** | **1** |  |
| i | The words artificial intelligence, AI, machine learning, deep learning, or other terminology related to artificial intelligence are reported in the title | Y | The study title indicates that a machine learning system is investigated [page 1]. |
| ii | The outcome of interest predicted by the AI model is reported in the title | Y | The study title indicates that the outcome of interest is prostate cancer-specific mortality [page 1]. |
| iii | The target population in which the AI model will be used is reported in the title | Y | The study title indicates that the target population involves non-metastatic prostate cancer patients [page 1]. |
| **Introduction** | | | |
| **2** | **Background: Describe the clinical problem and rationale for developing AI models. Review existing relevant literature exploring AI models for the problem being addressed. [Max score 1]** | **1** |  |
| i | The clinical context and rationale for developing/updating an AI model(s) to address the clinical problem are presented | Y | The authors describe how accurate prediction of prostate cancer-specific mortality may help identify patients who would benefit most from treatment. However, current predictive models are limited in that they either predict biochemical recurrence, which is a poor surrogate for survival, or fail to capture complex, non-linear relationships between variables. The authors propose the use of a novel machine learning framework on a large, national dataset to predict 10-year cancer-specific mortality in men with  non-metastatic prostate cancer [pages 1-2]. |
| ii | A synthesis of existing AI models that predict the same outcome is provided. If there are no existing models, this should be stated | Y | In the research in context, the authors found that only few machine learning studies examined prognostication in prostate cancer and were primarily based on small, single ethnic cohorts [page 2]. |
| **3** | **Objective and Problem: Clearly state what the proposed AI model(s)**  **aims to address with respect to the study population and outcome. [Max score 1]** | **1** |  |
| i | The objectives are presented | Y | The authors aimed to use a novel machine learning framework, Survival Quilts, on a large, national dataset  to predict 10-year cancer-specific mortality in men with non-metastatic prostate cancer. Secondly, they aimed to compare its performance against existing clinical models [page 2]. |
| ii | The target population and outcome of interest are stated | Y | The target population is non-metastatic prostate cancer patients. The outcome of interest is prostate cancer- specific mortality [page 2]. |
| **Methods** | | | |
| **4** | **Source of Data: Describe how the dataset was obtained (e.g., single/multi-center, local/national database, etc.), and study period. If relevant, the diversity of the dataset is also described (e.g., inclusion of community hospitals, low/middle income populations, and**  **institutions from other countries). [Max score 8]** | **8** |  |
| i | How many institutions were included in the dataset? | Multiple institutions | The dataset was based on the Surveillance, Epidemiology, and End Results (SEER) Program, a national  registry that provides epidemiologic information on cancer statistics and survival rates in the United States [page 2]. |
| ii | Was the study period (start and end dates) reported? | Y | The study period was from 01-Jan-2000 to 31-Dec-2016 [page 3]. |
| iii | Was the length of follow-up reported, if applicable? | Y | Length of follow-up was from date of diagnosis to time-to-event or date of last contact (either death or last follow-up) [page 3]. |
| iv | What was the setting(s) of the institutions included in the data or inferred based on their description?  *If not reported or unknown, select No.*  Academic institutions | Y | The SEER Program includes information from academic, community, and low-income patient populations [pages 2]. |
|  | Institutions from multiple (> 1) countries | Y | See explanation for item 4i [page 2] |
|  | Community-based or rural hospital(s) | Y | See explanation for item 4i [page 2] |
|  | Low/middle income patient populations | Y | See explanation for item 4i [page 2] |

| **5** | **Eligibility criteria: Specify all criteria for inclusion/exclusion of patients and features. Provide appropriate details (e.g., adults, age >**  **18) and rationale. [Max score 3]** | **3** |  |
| --- | --- | --- | --- |
| i | Inclusion criteria are provided | Y | All men with histologically confirmed non-metastatic prostate cancer (site code C61.9) in the specified study period (item 4ii) were included [page 3]. |
| ii | Exclusion criteria are provided | Y | Patients were excluded if 1) there was evidence of metastatic disease, 2) missing information on survival, PSA, Gleason grade, or stage, or 3) age < 35 or > 95 years [page 3]. |
| iii | Details and rationale for criteria are provided | Y | Complete data were required for PSA, Gleason grade, stage, and prostate cancer-specific mortality since they were required by all clinical models [page 3]. |
| **6** | **Ground truth: Define the ground truth of interest. Describe how it was collected (e.g., manual annotation by experts) and encoded (e.g.,**  **binary, categorical, dichotomized continuous, continuous variable, etc.). [Max score 6]** | **6** |  |
| i | Ground truth of interest is clearly defined  *For unsupervised learning, describe what measure(s) and associated data will be used to assess cluster validity (e.g., correlating disease- specific features with overall survival)* | Y | Prostate cancer-specific mortality [page 3] |
| ii | How was the ground truth determined? | Objective, well-captured  ground truth (e.g., in- hospital mortality) | Ground truth was determined using International Classification of Diseases codes, as determined from the SEER website [page 3]. |
| **7** | **Data abstraction, cleaning, preparation: Describe the methods used to develop the final dataset, with consideration of feature abstraction,**  **handling of missing data, feature engineering, and removal of features. [Max score 7]** | **4** |  |
| i | Rationale provided for choice of candidate features (e.g., based on prior research, clinical relevance, available data, etc.) | Y | Features were selected based on known predictors of prostate cancer-specific mortality [pages 1-3]. |
| ii | Time-windows for abstracted features are specified (e.g., vital signs recorded within the past 12 hours will be used to predict sepsis) | Y | Features were abstracted at the time of prostate cancer diagnosis [page 3]. |
| iii | How was missing data handled?  *If there is no missing data, it should be clearly stated that there is no missing data, select Not applicable. If it is unclear whether there is*  *missing data or how it was handled, select Not reported* | Explicit modeling of missing data without justification | Mean imputation [page 3] |
| iv | Transformation/Augmentation: Details provided for how data was altered to change its representation (e.g., normalization, log-transformation, one- hot encoding, image rotation, image translation, adjusting image contrast) *If not performed, it should be clearly stated that it was not performed, select Not applicable. If it is unclear whether it was performed or not*  *explicitly stated, select No* | N | Not specified |
|  | Modification/Cleaning: Details provided for how data was altered in a non-uniform manner (e.g., outlier removal).  *If not performed, it should be clearly stated that it was not performed,*  *select Not applicable. If it is unclear whether it was performed or not explicitly stated, select No* | N | Not specified |
| v | Outline any methods used to remove features (e.g., clinical judgement, principal component analysis, recursive feature elimination, correlation, or ablation analysis), if applicable  *If not performed, it should be clearly stated that it was not performed, select Not applicable. If it is unclear whether it was performed or not explicitly stated, select No.* | NA | Not applicable |
| **8** | **Data splitting: Specify how the data was divided into the training, validation, and testing cohorts. [Max score 7]** | **4** |  |
| i | What was the method of data splitting used? | Random split (i.e., random 80:20 train-test split) | Random 64:16:20 training/validation/testing split [page 3] |

| ii | What was the method used to evaluate model generalizability | Internal validation (i.e., separate cohort not used for model training from  the same institution) | The SEER dataset was used for both training, validation, and testing [page 3]. |
| --- | --- | --- | --- |
| iii | Were there any concerns of data leakage (i.e., data preprocessing performed prior to data splitting, training, and testing on the same data)? | N | No concerns for data leakage [pages 2-3]. |
| **9** | **Sample size calculation: Provide rationale for sample size required**  **for model development (e.g., based on power calculation). [Max score 5]** | **0** |  |
| i | Minimum sample size required reported | N | Not specified |
| ii | Minimum number of events required reported | N | Not specified |
| iii | Details provided for sample size calculation (e.g. assumptions for event rates, target performance, power, significance level). Can be provided in  supplementary material | N | Not specified |
| **10** | **Baseline: Describe the baseline model that will serve as a comparison for the AI model(s). [Max score 8]** | **6** |  |
| i | Existing model from prior literature used for comparison | Y | Cancer of the Prostate Risk Assessment score, Cambridge Prognostic Groups, National Comprehensive  Cancer Care Network, Genitourinary Radiation Oncologists of Canada, American Urological Association, European Association of Urology, National Institute for Health and Care Excellence [page 3] |
| ii | Regression model using same features in AI model used for comparison | N | Not specified |
| iii | Domain expert (e.g., clinician judgement) or current standard of care (gold standard) used for comparison | Y | PREDICT Prostate and Memorial Sloan Kettering Cancer Center nomograms are the most widely used models in clinical practice [page 3]. |
| **11** | **Model description: Describe the AI model(s) and software libraries investigated. [Max score 2]** | **2** |  |
| i | Type of AI model(s) reported (e.g., random forest, support vector machine, convolutional neural network) | Y | Survival Quilts, Cox regression, random survival forest, conditional inference survival forest, DeepHit [page 3, appendix page 3] |
| ii | Software libraries reported (e.g., scikit-learn 1.1.2) | Y | Scikit-learn 3.6.5, R 3.6.1, Python 3.6.5 [page 3. appendix page 3] |
| **12** | **Hyperparameter tuning: Specify all model hyperparameters that were optimized, the search space for hyperparameter tuning, and evaluation metric(s) used to optimize parameters. Details can be included in Supplementary Material. [Max score 5]** | **2** |  |
| i | Hyperparameters that are tuned are listed (e.g., number of trees, max depth, number of neurons) | N | Not reported |
| ii | Optimization metric is specified (e.g., accuracy, AUROC, etc.) | Y | C-index [page 3] |
| iii | Hyperparameter search strategy is described (e.g., random-, grid-search, etc.) | Y | Grid-search [page 3] |
| iv | Search space for hyperparameters are provided | Not reported | Not reported |
| **Results** | | | |
| **13** | **Cohort characteristics: Provide the total cohort size and summary statistics of the training, validation (if used), and testing cohorts,**  **including incidence of the ground truth of interest. [Max score 4]** | **4** |  |
| i | Total cohort size, number of samples with missing data, and follow-up time (if applicable) are reported | Y | Table 1 [page 4] |
| ii | Summary statistics of each cohort provided to show similarities and differences among cohorts | Y | Table 1 [page 4] |
| iii | Incidence of ground truth(s) of interest is reported | Y | Table 1 [page 4] |
| **14** | **Model specification: Present the final AI model(s) and specify the final panel of features included and hyperparameters tuned. Final hyperparameters can be listed in Supplementary Material. [Max**  **score 3]** | **2** |  |
| i | Type of AI model(s) is reported | Y | Models were specified in both the Methods and Appendix [page 3, appendix pages 1, 3]. |
| ii | Final set of features are reported | Y | Age, PSA, biopsy core involvement, T stage, Gleason Group Group [appendix page 2] |
| iii | Final set of hyperparameters are reported | N | Not reported |

| **15** | **Model evaluation: List the evaluation metrics used to assess performance and calibration, including the justification for selection.**  **[Max score 5]** | **5** |  |
| --- | --- | --- | --- |
| i | Measure(s) for model discrimination is reported (e.g., AUROC, AUPRC, c-index, etc.)  *If multiple measures of discrimination are provided and at least one*  *includes a measure of statistical significance, select Measure(s) with statistical significance* | Measure(s) with statistical significance (e.g., confidence  interval, standard error, p-value) | Time-dependent c-index with 95% confidence intervals determined using 10,000 bootstrap samples (Table 2) [page 3, 5] |
| ii | Rationale provided for which metric is most clinically relevant for the problem at hand | Y | Time-dependent c-index was used to assess discrimination at the 10-year timepoint [page 3]. |
| iii | Measure(s) for model calibration is reported (e.g., calibration plots, calibration slope and intercept)  *If both calibration plot and statistical summary of calibration are provided, select Calibration plot* | Calibration plot | Brier scores (Table 2), calibration plot (Figure 2) [page 5] |
| **16** | **Clinical utility assessment: Describe appropriate metrics for readers to understand the risk/benefit trade-offs of using the AI model at the specified decision threshold (e.g., decision curve analysis). [Max score 5]** | **5** |  |
| i | Measure(s) of clinical utility is reported  *If both sensitivity or specificity for a specified threshold and decision curve analysis are provided, select Decision curve analysis* | Decision curve analysis | Figure 3 [page 6]. |
| **17** | **Bias assessment: Compare evaluation metrics for the AI model(s) and reference standard when stratified by patient- and task-specific subgroups to identify subgroups that benefit, are not helped at all, or harmed by the models.**  **Patient-specific subgroups may include age group, gender, ethnicity, or socioeconomic status.**  **Task-specific subgroups are disease-specific and may include risk stratification (e.g., low-, intermediate-, and high-risk disease in**  **prostate cancer), or subtyping (e.g., different bacteria in positive blood cultures). [Max score 6]** | **2** |  |
| i | Patient-specific: Performance (e.g., AUROC) is evaluated across at least one subgroup | Y | Age < 65 vs ≥ 65 years (Table 3) [page 5] |
| ii | Patient-specific: Clinical utility (e.g., sensitivity or specificity for a specified threshold) is evaluated across at least one subgroup | N | Not performed |
| iii | Patient-specific: More than one subgroup is evaluated in either performance or clinical utility | Y | White vs Black vs Other (Table 4) [page 6] |
| iv | Task-specific: Performance (e.g., AUROC) is evaluated across at least one subgroup | N | Not performed |
| v | Task-specific: Clinical utility (e.g., sensitivity or specificity for a specified threshold) is evaluated across at least one subgroup | N | Not performed |
| vi | Task-specific: More than one subgroup is evaluated in either model performance or clinical utility | N | Not performed |
| **18** | **Error analysis: Analyze predictive errors to identify characteristics that are more prone to inaccurate predictions. Determine if there are**  **any surprise errors (e.g., clearly inaccurate predictions based on clinical judgement). [Max score 4]** | **0** |  |
| i | Analysis of predictive errors is reported | N | Not performed |
| ii | Analysis of surprise errors is reported | N | Not performed |
| **19** | **Model explanation: Describe methods used to explain AI models (e.g., SHAP, LIME, Grad-CAM) [Not scored]** | **0** |  |
| i | Model explanations are provided | N | Not performed |
| **Discussion** | | | |
| **20** | **Critical analysis: Describe main findings and limitations of the study. [Max score 5]** | **5** |  |

| i | An overall interpretation of the results is presented, which may include:   - New predictors of the ground truth of interest discovered using AI - Strengths of the AI model(s) compared to current models in the literature - Why the AI model(s) performed better/worse than what is currently available? - (Optional) If feature importance rankings were used, describe whether they were aligned with clinical intuition and known prognostic factors | Y | The authors highlight that this is the first study using the SEER database to predict prostate cancer-specific mortality. This was also the first application of their novel machine learning framework, Survival Quilts, which outperformed current nomograms. They argue that clinicians should move away from tier-based to multivariable models [pages 5-7]. |
| --- | --- | --- | --- |
| **21** | **Implementation into clinical practice: Describe how the AI model(s) can be applied to clinical practice, with respect to the potential to improve patient care, clinical decision-making, and/or efficiency.**  **[Max score 1]** | **1** |  |
| i | Potential application(s) to clinical practice and future directions are discussed | Y | The authors outline how their model can be implemented to predict 10-year prostate cancer-specific mortality [page 7]. |
| **22** | **Limitations: Discuss the limitations of the AI model(s), with consideration of the data, features, model(s), and/or biases. [Max score 2]** | **2** |  |
| i | Limitations are discussed | Y | The authors outline limitations including 1) a heavily skewed population to earlier stage disease due to regular PSA screening, 2) few death events and limited follow-up, 3) potential bias in the dataset, 4) large amount of missing data for biopsy core involvement, 5) inability to examine the effects of treatment and comorbidities on cancer-specific mortality, 6) the lack of prostate MRI and molecular markers, and 7)  exclusion of metastatic prostate cancer patients [page 7]. |
| **Other Information** | | | |
| **23** | **Disclosures: Disclose all financial relationships, sources of funding, and potential conflicts of interest. [Max score 1]** | **1** |  |
| i | All relevant disclosures are reported | Y | No funding source or competing interests [pages 3, 7] |
| **24** | **Transparency: Share the data, data dictionary, source code, or release an application that runs the code. [Max score 10]** | **6** |  |
| i | Data dictionary: A description is provided for all features and ground truth, with consideration of the following:   - Data type (i.e., categorical or numerical) - Method of collection or measurement (e.g., serum hemoglobin in g/dL) - Range of values (e.g., yes or no, 0.5-250 g/dL) | N | Not provided |
| ii | Data availability: How can other researchers access the data used in the study?  *Data availability needs to be explicitly stated to receive points* | Available on an established data sharing repository (e.g., MIMIC) | The data sharing statement indicates that the SEER database is publicly available in the national registry [page 7]. |
| iii | Model availability: How can other researchers access the model(s) used in the study?  Nomogram/scoring system/website available to use model for single predictions | N | Not provided |
|  | Trained model available to generate prediction in bulk (i.e., from a  dataset) | N | Not provided |
|  | Complete source code available | N | Not provided |
|  | Executable end-to-end (e.g., dependency file, documentation on how to run the code) available | Y | Data sharing statement includes a Github repository to their Survival Quilts model with documentation on how to run their code [page 7]. |
|  | | | |
|  | **Overall APPRAISE-AI score (out of 100)** | **71** | |
|  | **Quality based on overall APPRAISE-AI score** | **High** | |
|  | **Clinical Relevance (out of 4)** | **4** | |
|  | **Data Quality (out of 24)** | **21** | |
|  | **Methodological Conduct (out of 20)** | **10** | |
|  | **Robustness of Results (out of 20)** | **12** | |
|  | **Reporting Quality (out of 12)** | **12** | |
|  | **Reproducibility (out of 20)** | **12** | |
